# Evaluating the role of salt intake in achieving WHO NCD targets in the Eurasian Economic Union: A PRIME modeling study

**DOI:** 10.1371/journal.pone.0289112

**Published:** 2023-07-21

**Authors:** Vern Perera, Luke N. Allen, Clare Farrand, Edwin Jit Leung Kwong, Isurujith Liyanage, Kremlin Wickramasinghe

**Affiliations:** 1 Faculty of Medical Sciences, University of Sri Jayewardenepura, Colombo, Sri Lanka; 2 Clinical Research Department, London School of Hygiene and Tropical Medicine, London, United Kingdom; 3 World Health Organization Regional Office for Europe, Copenhagen, Denmark; 4 Melbourne School of Population and Global Health, University of Melbourne, Carlton, Australia; 5 WHO European Office for the Prevention and Control of Noncommunicable Diseases (NCD Office), Moscow, Russian Federation; Universidade de Sao Paulo, BRAZIL

## Abstract

The World Health Organization has set clear global targets in reducing non-communicable disease mortality by 2030 in its sustainable development goals. This study models the number of deaths that could be averted if Eurasian Economic Union (EEU) member states met the target of reducing their population’s current mean salt intake by 30% to achieve mortality reduction targets. Using the WHO *Preventable Risk Integrated ModEl* (PRIME), we modelled the mortality impact of reducing salt consumption by 30%, as well as according to WHO recommended levels (5 g/person/day), for the five member states of the EEU. PRIME models the number of averted deaths from reducing salt intake by applying established risk ratios to a given population. The baseline demographic and mortality data that are required to generate these estimates were obtained from the relevant government statistical bodies, and salt intake data were referenced from surveillance studies. Uncertainty intervals were generated using Monte Carlo simulation. If salt consumption was reduced by 30%, we estimate that there would have been 94,150 (95%UI: 47,329 to 137,131) fewer deaths due to cardiovascular disease in the EEU in the baseline year, with males and the elderly being more affected. If the WHO-recommended maximum salt intake of 5 g/day was achieved, a total of 193,155 (95%UI: 98,548 to 272,536) deaths would have been prevented. These findings underline the importance of incorporating effective policy changes to meet targets in reducing NCD mortality by one-third by 2030.

## Introduction

Non-communicable diseases are the leading cause of death globally, which are responsible for over 70% of all deaths, with most occurring in those over 50 years of age in the WHO European region [1, 2]. A majority of these deaths may be prevented by addressing their associated risk factors, such as salt intake, which contributes to cardiovascular disease (CVD) and stroke–the conditions representing the largest proportion of NCD deaths [3]. As NCDs place a significant burden on all European countries’ economies, health systems, and citizens’ well-being, reducing NCD risk factors should be a policy priority. Although a decline in NCD mortality has been observed in the last 20 years in the WHO European region, this region continues to be the most heavily burdened by NCDs [4]. The Eurasian Economic Union (EEU), which is comprised of the member states Armenia, Belarus, Kazakhstan, Kyrgyzstan, and the Russian Federation, have been shown to have high levels of hypertension and cardiovascular disease partly driven by very high levels of sodium intake, as demonstrated by various surveillance studies [5–14]. Specifically, 6.04% of all CVD deaths have been shown to be attributed to high sodium intake in the EEU [15].

In 2015, all 194 WHO Member States universally endorsed the ‘Sustainable Development Goals’ (SDGs), which are a set of 17 goals with 169 targets created to ensure a sustainable future for all countries by 2030 [16]. SDG 3.4 highlights interventional goals to reduce premature NCD mortality by one third. To achieve this, the World Health Assembly (WHA) has outlined multiple targets in the Global Monitoring Framework, including reducing mean salt intake by 30% by 2025 [17]. No formal baseline year was established in monitoring its progress, so countries are implicitly encouraged to use their latest available salt consumption estimate as the baseline. The WHO European region is currently on track to meet the overarching target of reducing overall mortality from major NCDs, such as cardiovascular diseases, cancer, diabetes, or chronic respiratory disease [17]. However, the recommended reductions in salt intake are unlikely to be met, with the latest data showing that an overwhelming majority of WHO European countries far exceed recommended salt intake levels [18, 19].

Multiple risk factors are known to be attributed to CVD mortality, such as smoking, reduced physical activity, and diabetes. However, salt, an easily incorporable driver of sodium in the diet, is a highly modifiable risk factor known for its role in hypertension [3, 20]. Hence, doing away with excess salt consumption could significantly reduce the risk of hypertension-mediated CVD mortality. Targeting salt intake as a sole risk factor in modelling its effect on CVD mortality could therefore provide interesting context and the necessary background to start or accelerate salt reduction initiatives in the EEU, where progress is currently lacking. Thus, by using the WHO “Preventable Risk Integrated ModEl” (PRIME), a public-access retrospective scenario modelling tool developed by researchers at the University of Oxford’s *WHO Collaborating Centre on Population Approaches for NCD Prevention*, this study aims to estimate the number of deaths that would have been averted had the relevant countries’ salt intake levels been reduced by 30% in order to achieve SDG 3.4 [21].

As all of the EEU countries would still have a per capita salt consumption that exceeds WHO-recommended limits even with a 30% reduction, we also set out to model the impact of reducing population-level salt intake down to the WHO recommended maximum threshold of 5 g/day [22].

This work was commissioned to quantify the scale of the problem as the next step in developing robust salt reduction programs at the national level for EEU states.

### Context: Overview of salt intake and policy in the EEU

#### Armenia

According to the latest available data, Armenia does not have policies for salt reduction, and most of the existing diet-related policies are concerned with correcting undernutrition rather than addressing NCDs [23]. As a result, salt intake among the population is largely unregulated. According to the 2016 WHO ‘STEPS’ NCD risk factor survey, the mean per capita salt intake is 9.8 g/day, almost double the WHO recommended levels of 5 g/day [5].

#### Belarus

Belarus has adopted measures to reduce salt consumption in diet, primarily through food reformulation [24]. However, the 2016 STEPS survey found the mean total salt intake to be 10.6 g/day [6].

#### Kazakhstan

The consumption of foods high in salt is commonplace in Kazakhstan, which is the world’s leading consumer of salt with a mean daily intake of 17.24 g/day, according to two cross-sectional surveys from 2016 [7]. A number of studies have indicated that salt reduction would result in large returns of investment in preventing NCD mortality in Kazakhstan; however, no national targets have been set [25].

#### Kyrgyzstan

Unfortunately, no data are available on per capita salt consumption. However, FeedCities, a WHO project, has found that food in public marketplaces in the capitol have very high levels of salt [26]. Currently, Kyrgyzstan has no specific policies dedicated to reducing salt consumption, however, its national targets align with that of the recommended WHO salt intake levels [23]. Furthermore, FeedCities has suggested that the government uses proxy measures (such as measuring the amount of salt added to food) to determine the country’s progress toward its national salt targets—although we note that adopting a gold-standard measure of salt surveillance would be far more accurate [26]. Without reliable data on salt intake, it will be impossible to monitor progress towards the country’s targets.

#### The Russian Federation

As a member of the European Salt Action Network, Russia has taken steps to reduce salt intake and prevent NCD mortality within its population [23]. Nevertheless, a survey on the dietary structure of its population in 2018 showed that the mean total salt intake was 14.87 g/day, almost triple the recommended WHO level [8].

## Materials and methods

### The PRIME modelling tool

In this study, we used the ‘Preventable Risk Integrated ModEl’ (PRIME) recommended by the WHO for modelling the impact of hypothetical changes to NCD risk factors. We used PRIME to determine the number of deaths that could have been prevented from reducing salt intake at the population level in each of the EEU countries. PRIME is an open-access scenario modelling tool that can estimate the influence of changing NCD risk factors on mortality for a given year against counterfactual scenarios, according to data fed from meta-analyses [21, 27]. PRIME only works retrospectively, modelling the number deaths that would have been averted given a counterfactual risk factor distribution. PRIME requires three main inputs: the demographic distribution of salt intake across the population for a given year; the demographic distributions of deaths for the same year; and a counterfactual distribution of salt intake.

For salt intake, PRIME utilizes the findings of a meta-analysis in its parametrization of associated averted deaths, where lowering salt intake by 6 g/day brought about a reduction of 5.88 mmHg in systolic blood pressure (SBP) [28]. Importantly, this study found that these findings were significant regardless of gender or ethnicity, though its effect on specific global populations cannot be directly elucidated. However, more recent meta-analyses and systematic reviews have also shown comparable levels of reductions in SBP following salt intake reduction strategies. Salt substitution and nutritional awareness campaigns were shown to produce a reduction of 7.44 mmHg in SBP, while salt substitution alone resulted in a reduction of 4.61 mmHg and 4.80 mmHg SBP overall in two other studies, regardless of geographical location or population demographics [29–31]. These studies further recommended the use of multi-component strategies in reducing sodium salt intake to prevent associated CVD mortality, which will be discussed further.

All of the estimated averted deaths due to reductions in salt intake in PRIME are the result of reduced blood pressure-related cardiovascular deaths, which is important to note as further reductions in deaths due to lowered salt intake from other mechanisms are not accounted for.

### Setting up PRIME with salt intake as the sole risk factor

An empty PRIME model was downloaded from the toolkits section of the WHO Regional Office for Europe website [32]. The salt intake data for each EEU member state (Armenia, Belarus, Kazakhstan, and Russia) were adopted from the most recent national surveillance studies from each country, which are tabulated in Table 1 [4–8]. There are no available data on salt intake for Kyrgyzstan as national level studies have never been conducted. Therefore, following discussion with the WHO European NCD Office, we decided to use Kazakhstan’s salt intake data as a proxy based on the fact that Kazakhstan is the most similar country for which data were available. In addition, there are no standard deviation (SD) data available for Russia as the population size in the salt intake analysis was not reported, and salt intake values according to gender were not available [8]. Based on discussion with the WHO European Office for the Prevention and Control of Noncommunicable Diseases, it was decided that the salt consumption of Belarus’ was the most closely matched, so the SD of male Belarus salt intake were used for Russia.

**Table 1:** Mean salt intake and counterfactual scenarios for each EEU member state.

Country	Mean Salt (g/day) *Female*	Mean Salt (g/day) *Male*	30% Reduction in Salt Intake (g/day) *Female*	30% Reduction in Salt Intake (g/day) *Male*	WHO Recommended Salt Intake (g/day) *Female and Male*	SD *Female*	SD *Male*
**Armenia (2018)**	8.4	11.0	5.88	7.7	5.0	2.06	2.06
**Belarus (2018)**	9.0	12.4	6.3	8.68	5.0	2.48	3.14
**Kazakhstan (2017)**	16.14	18.51	11.30	12.96	5.0	7.48	8.63
**Kyrgyzstan (2016)**	16.14	18.51	11.30	12.96	5.0	7.48	8.63
**Russia (2019)**	14.87	14.87	10.41	10.41	5.0	3.14	3.14

The counterfactual values for salt intake used in this study were the 30% reduction in each country’s known salt intake for the latest year where data were available. Standard deviations for the counterfactual scenarios were equal to the baseline SDs as they were assumed to be the same proportion of the mean. In our secondary analysis, we used the WHO recommended 5g daily intake for all age groups and genders. Population and NCD mortality data were taken from each country’s official statistical body and published studies, respectively [4, 33–37]. The population data were taken from the same year as the latest available mortality data for better accuracy. The corresponding PRIME models were then established for each EEU member state after inputting baseline salt intake, counterfactual values, as well as the population and mortality data. S1–S6 Tables provide the population and mortality data for each EEU member state that were used in the analysis.

### Estimating the number of averted deaths due to reductions in salt intake

After the corresponding data were entered into each country’s PRIME model, a Monte Carlo analysis was performed within PRIME for 5,000 iterations. PRIME uses Monte Carlo simulations to estimate risk according to each risk factor’s confidence intervals [27]. In this study, PRIME used the 95% confidence intervals from a meta-analysis (2.5 to 9.2) in estimating the number of averted deaths from reduced salt intake in its Monte Carlo analysis [28]. After the analysis for each country was complete, the number of averted deaths and its associated uncertainty intervals were generated. PRIME also provides a breakdown of prevented deaths according to disease, which is elaborated further in the following section.

Table 1 summarizes the population means for each baseline year, plus the counterfactual values. It should be noted that salt intake was not reported according to age banding in all countries; thus, the same level of salt intake was assumed for each age group.

## Results

### Achieving salt intake intervention goals

Table 2 summarises the estimated reduction in deaths observed in each country. Overall, if the EEU achieved the goal of a 30% reduction in salt intake, we estimate that a total of 94,150 (95%UI: 47,329 to 137,131) deaths due to cardiovascular disease would have been prevented across the region in the years for which we have data. The greatest overall gains were observed among the elderly (age ≥ 65) (68,193/94,150; 72.43%) and male (47,102/94,150; 50.03%) populations. Slightly more female than male deaths were prevented in Russia (42,290/82,991; 50.96%), whereas more male deaths were prevented in each of the other countries and overall. Among the elderly, more female (40,117/68,193; 58.83%) than male (28,076/68,193; 41.17%) deaths were prevented, which was also observed within each EEU state.

**Table 2:** Total number of prevented deaths according to different interventions on salt intake.

Country and baseline year	Deaths prevented with a 30% reduction in salt intake (% of all deaths prevented) 95%UI	Deaths prevented by adopting WHO recommended levels (% of all deaths prevented) 95%UI
**Armenia**	**839 (5.55%)**	**1,315 (8.69%)**
2018	344 to 1,292	595 to 1,982
**Belarus**	**4,924 (6.17%)**	**8,500 (10.65%)**
2018	2,054 to 7,620	3,664 to 12,830
**Kazakhstan**	**3,515 (5.75%)**	**8,034 (13.15%)**
2017	1,506 to 5,209	3,892 to 11,371
**Kyrgyzstan**	**1,881 (8.54%)**	**4,339 (19.69%)**
2016	852 to 2,824	2,052 to 6,237
**Russia**	**82,991 (7.81%)**	**170,967 (16.08%)**
2019	36,616 to 126,144	77,467 to 250,997
**Total**	**94,150 (7.58%)**	**193,155 (15.56%)**
	47,329 to 137,131	98,548 to 272,536

**Fig 1:**
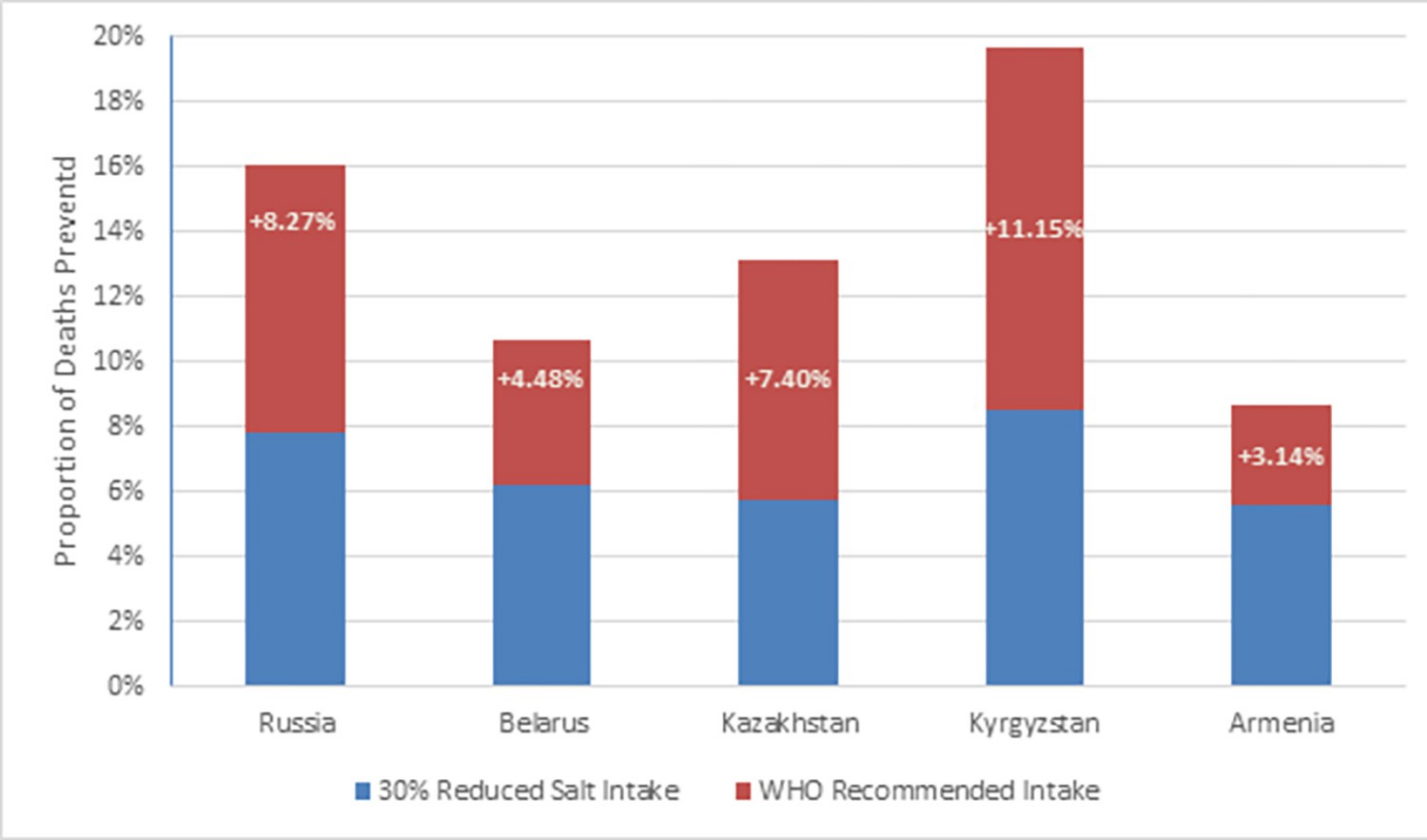
Total deaths prevented after salt intake interventions. Further reductions in salt intake resulted in an even higher proportion of deaths averted. Kyrgyzstan had more than double the number of deaths prevented after adopting the WHO recommended salt intake compared to a 30% reduction.

The WHO has recommended that adults consume a maximum of 5 g salt per day [22]. If this level of intake was achieved, a total of 193,155 (95%UI: 98,548 to 272,536) deaths would have been prevented across the EEU in the years for which we have data. Fig 1 provides an overview of the effect of different levels of salt intake interventions on mortality in the EEU. As shown in the figure, Kyrgyzstan benefited the most from reducing salt intake at both levels of intervention, where 8.54% and 19.69% of deaths were prevented at 30% reduced salt intake and at WHO recommended levels, respectively, compared to if no action was taken in reducing salt consumption. In the EEU overall, nearly 16% of cardiovascular deaths would have been prevented if the WHO threshold was adhered to.

### Reduced salt intake drove a significant proportion of CVD deaths down

**Fig 2:**
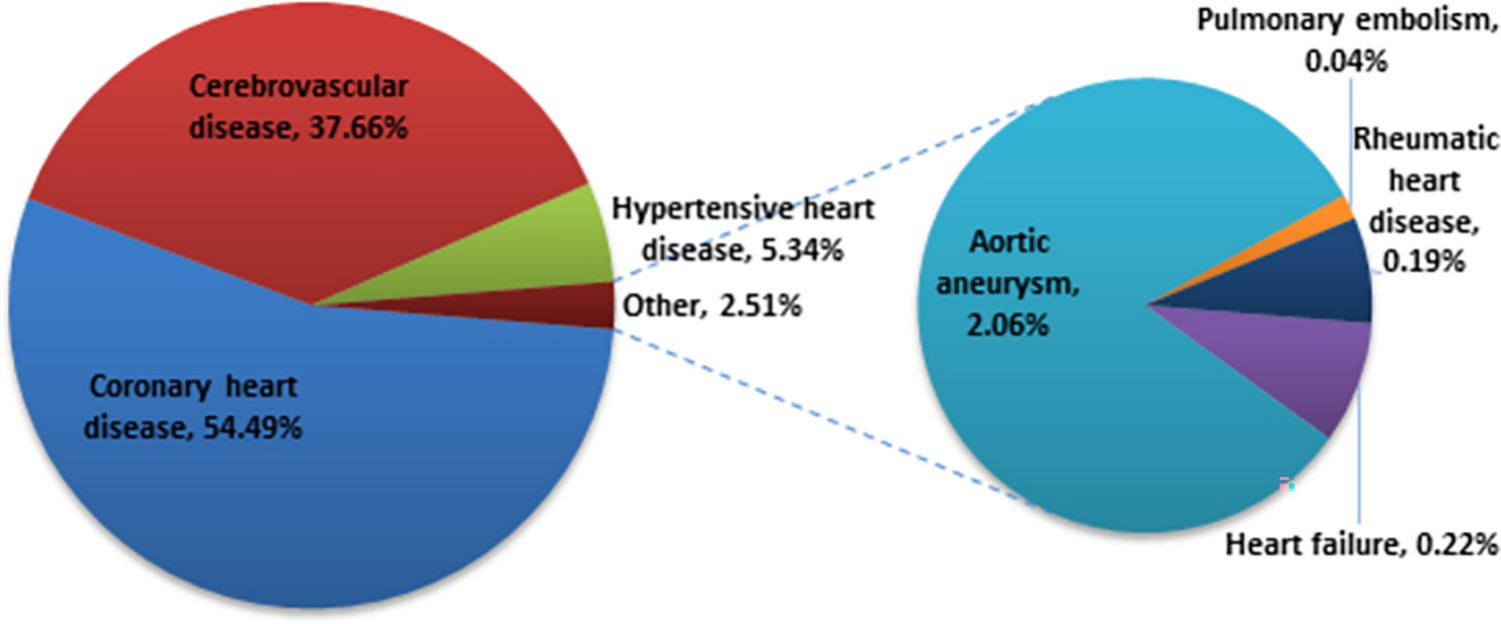
Proportion of cardiovascular deaths prevented following a 30% reduction in salt.

As shown in Fig 2, among all of the estimated averted deaths, coronary heart disease (51,301/94,150; 54.49%), cerebrovascular disease (35,460/94,150; 37.66%), and hypertensive heart disease (5,023/94,150; 5.34%) were the most prevented CVD deaths following a 30% reduction in salt intake. Nearly the same proportion of averted deaths were observed after adjusting salt intake to 5 g/day. The numerical breakdown of CVD deaths generated by PRIME as a result of interventions in salt intake is provided in S7 and S8 Tables.

### Hypertensive heart disease benefited the most following intervention

By examining changes in individual CVD deaths, it is possible to observe trends in case-specific mortalities. Although coronary heart disease was the most commonly prevented cause of death following a 30% salt reduction, hypertensive heart disease experienced the largest relative reduction (5,023/18,522; 27.12%). In females, mortality from hypertensive heart disease (2,905/10,772; 26.97%), aortic aneurysm (1,024/8,598; 11.91%), and cerebrovascular disease (18,790/171,172; 10.98%) saw the greatest reductions compared to their individual baselines. A similar pattern was observed in males, with hypertensive heart disease (2,118/7,750; 27.33%), cerebrovascular disease (16,670/119,897; 13.90%), and aortic aneurysm (915/7,669; 11.93%) being the most reduced.

**Fig 3:**
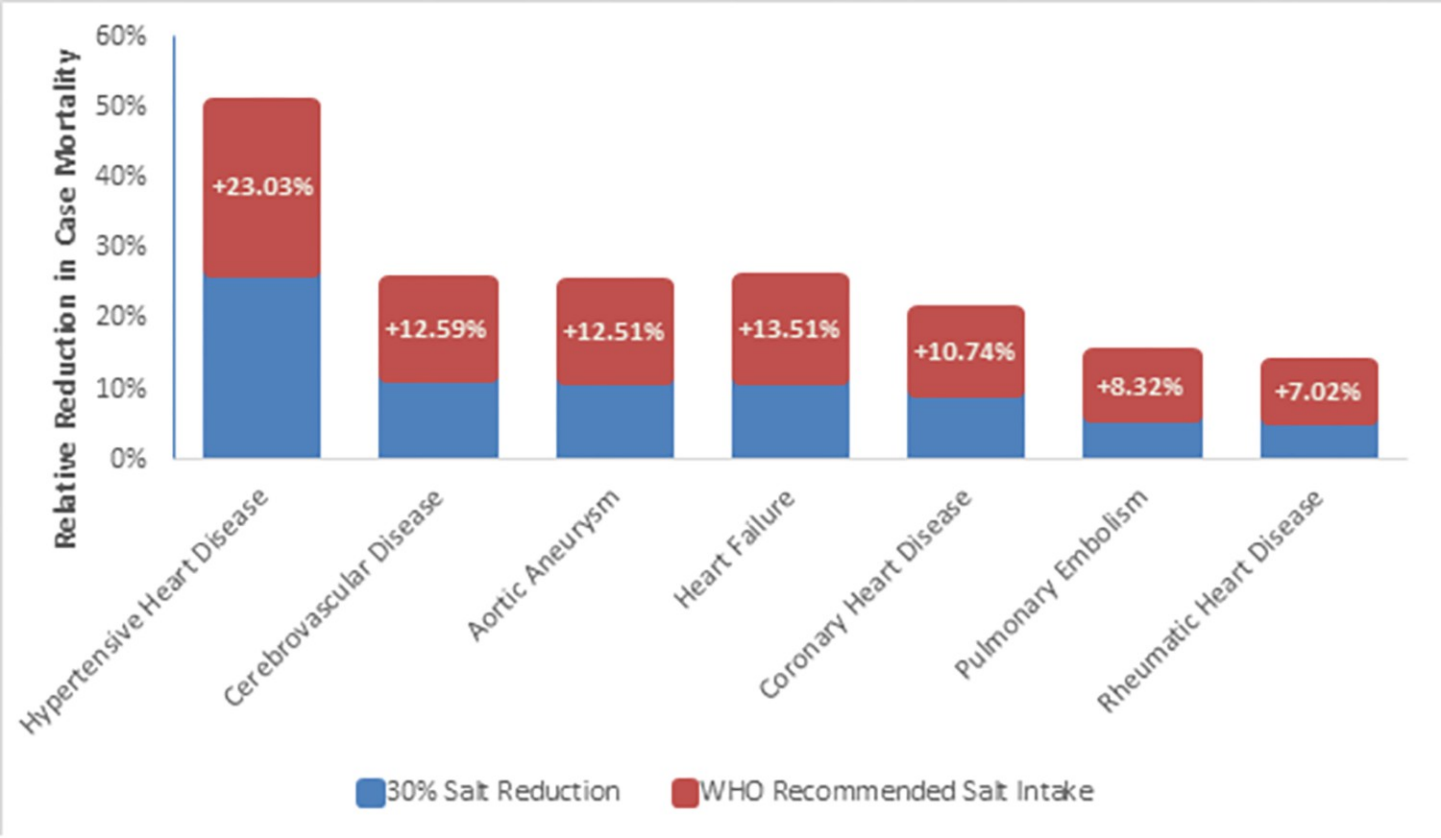
Relative reductions in disease-specific mortality after adopting salt intake interventions.

By further restricting salt intake to 5 g/day, we estimated that there would have been 50.15% (9,289/18,522) fewer deaths due to hypertensive heart disease in the EEU, almost double that of the previous level of intervention. In females, 49.55% (5,337/10,772) fewer deaths due to hypertensive heart disease were estimated with this level of salt intake, followed by 24.40% (2,098/8,598) and 22.37% (38,250/170,862) fewer deaths from aortic aneurysm and cerebrovascular disease, respectively. In males, 50.99% (3,952/7,750) of deaths due to hypertensive heart disease was reduced, while cerebrovascular disease (33,846/119,897; 28.23%) and aortic aneurysm (1,877/7,669; 24.48%) were the next most reduced, which was similar to the previous level of salt intervention. An overall breakdown of the reduction in case mortality is depicted in Fig 3.

### Premature deaths were markedly prevented following salt reduction

Following a 30% reduction in salt intake, 27.57% (25,957/94,150) of deaths in the working population (ages 15–64) were prevented throughout the EEU, among which males made up 73.30% (19,027/25,957) of all premature deaths. Notably, Kazakhstan had the largest reduction in premature deaths (1,552/3,515; 44.15%), followed by Kyrgyzstan (617/1,881; 32.80%), Belarus (1,369/4,924; 27.80%), Russia (22,204/82,991; 26.75%), and Armenia (215/839; 25.66%). A similar picture was seen after further reducing salt intake to 5 g/day, where 27.37% (52,899/193,155) of premature deaths were estimated to be prevented in the EEU, approximately double the number of deaths prevented compared to the previous level of intervention. Moreover, proportions when ranked by country and gender were again similarly observed at this level of intervention.

In this study, salt intake data was not available according to age; therefore, all ages were assumed to consume the same amount of salt per day. In addition, PRIME does not have data on the relative risk for CVD from salt consumption by age and instead assumes the same relative risk in all age groups. Hence, these findings may not be accurately representative of age.

## Discussion

Using the validated and WHO-endorsed PRIME modelling tool, we estimate that a total of 94,150 (95%UI: 47,329 to 137,131) and 193,155 (95%UI: 98,548 to 272,536) deaths would have been prevented in the EEU if member states reduced their population’s salt intake by 30% and to 5g per day, respectively. In all member states except Russia, males were more affected than females, and larger gains were observed in the elderly population compared to those under 65 years of age. In our PRIME analysis, Kyrgyzstan saw the largest benefits from reducing salt consumption, with 8.54% (1,881/22,038) and 19.69% (4,339/22,038) of deaths prevented after adopting each respective level of salt intervention. This reflects the fact that Kyrgyzstan has the highest baseline salt consumption.

Since hypertension is known to mediate CVD mortality, changes in salt intake have a major impact on hypertension-associated diseases, such as coronary heart disease, cerebrovascular disease, and hypertensive heart disease, as described in the findings [20, 38]. As demonstrated by this study’s findings, out of all of the estimated averted deaths in the EEU, coronary heart disease was the most prevented, while hypertensive heart disease mortality had the largest relative reduction across both levels of salt intervention, followed by cerebrovascular disease and aortic aneurysm.

### Reductions in premature deaths highlight the importance of salt consumption

Although this study analysed the sole effect of reduced salt intake in the EEU, the significance of salt consumption in averting CVD mortality can be appreciated, as demonstrated by the findings. A notable number of deaths were prevented among the working population throughout the EEU at both levels of salt intake intervention. This metric is particularly important as premature deaths may incur significant health and economic burdens on the population. For example, an Australian study that modelled the present value of lifetime earnings against CVD mortality found that $2.69 billion in earnings was lost due to premature deaths [39]. In Russia, which saw the largest number of averted deaths in this study, overall CVD mortality has been estimated to cost over €24 billion annually [40]. Though our analysis only modelled the impact of salt reduction on CVD deaths, its effect on premature population mortality was shown to be significant, which can provide a meaningful context for future cost analysis studies. Furthermore, as EEU member states have not reported age-banded data on salt intake, it is vital to adjust data collection methods to account for this gap in order to drive more focused policymaking.

### Patterns of salt consumption in the EEU

Dietary salt is a major component of nutrition in the EEU, particularly as the introduction of national iodization policies have contributed to widespread access to salt-based iodine in foods [41–43]. In Armenia, up to 80% of dietary salt intake stems from processed food consumption, with 31% of the population always or often consuming processed foods with high salt content, such as bread, meat, and pickled vegetables [5, 44]. Similarly, in Belarus, 28.5% of respondents in the 2016 STEPS survey reported to always or often eat processed food, including smoked meat, canned food, and salted snacks [6]. Kazakhstan is currently believed to be the world leader in salt consumption, having a mean salt intake of 17.24 g/day [7]. Among all sources of dietary salt, the highest levels of salt can be found in homemade food items, such as lagman and plov, both individually having more salt content per serving than the WHO recommended daily amount of 5 grams [25]. Moreover, in a study conducted by the Kazakh Academy of Nutrition, most respondents stated that they were unaware salt can cause hypertension, and less than 15% considered their perceived level of salt consumption to be too high [25]. Kyrgyzstan has not conducted salt surveillance studies for its population; therefore, it is impossible to gauge the effectiveness of its national salt reduction targets. However, marketplaces are known to carry food with high salt content, which may be indicative of average household consumption, though specific studies on this matter are lacking [26].

Following each country’s achievement of universal salt iodization, access to salt and salty products has been made commonplace, as mentioned above. As a result, most EEU countries have met or surpassed their iodization targets, and families have become accustomed to consuming salt-rich foods. Interestingly, Aslanyan et al. modelled the impact of salt reduction on iodine intake in Armenia and found that a 30% reduction in salt intake can sustain optimal levels of iodine consumption in adults [44]. Salt reduction strategies may not necessarily reverse or hinder the achievements of universal iodization, especially in countries with already high levels of salt intake, though further studies should be conducted to more accurately determine this. In a WHO report, potential for synergizing both iodine deficiency elimination and salt reduction programmes was highlighted, which included suggestions for policy and interventional changes, such as potassium-enriched (low sodium) salt, though further trials are needed to assess its stability as a driver of iodine and any adverse long-term health outcomes [45]. As salt intake is reduced, the harmonization of both interventions may require the gradual adjustment of levels of iodine fortification.

### Policy interventions are urgently required

Among the evaluated EEU countries, none are currently on track to meet the WHA target of a 30% reduction in salt intake by 2025, which will likely reflect in continually high preventable deaths rates from hypertension-associated diseases. As a result, a concerted effort in implementing national strategies to control salt intake in the population are urgently needed to prevent further deaths in the population. However, member states that have set targets for salt reduction may not have the corresponding data on salt intake, such as Kyrgyzstan, which is necessary to monitor the progress and eventual achievement of salt reduction goals. The Coordination Council on Public Health, a Kyrgyzstani government initiative responsible for high-level coordination of public health, has had its work on nutritional policymaking stalled due to changes in government [26]. This reflects that though changes in political landscapes may occur, all political stakeholders should ensure that such changes do not affect the planning and delivery of health policies. Meanwhile, NCD action plans are being coordinated through the Kyrgyzstan Health Ministry with the strong support of various NGOs, though specific plans for salt reduction do not currently exist.

As the most populous country in the EEU, Russia experienced the largest numbers of deaths prevented from salt intake intervention according to our estimations using Belarus’ male SD data as a proxy. Hence, ensuring the completeness of salt surveillance data will serve to better drive related policymaking and prevent a large proportion of deaths in the EEU. In addition, WHO NCD ‘STEPS’ surveillance studies have highlighted that public knowledge on the effects of increased salt intake are inadequate. Accordingly, countries that put forward population-wide salt reduction policies may have more successful outcomes from increased public education on salt consumption.

Targeted solutions to control salt intake, such as trade regulation and taxation, can be considered when designing national policies [2, 26]. However, designing and implementing an effective strategy at the national level can be challenging, especially for countries in low resource settings. The WHO has published a country support package to facilitate salt intake reduction for member states in the WHO European Region, which provides simple, affordable, and evidence-based options for carrying out population-wide salt intervention policies and would directly benefit the populations examined in this study [46]. The SHAKE technical package, one of the WHO’s pillars of salt reduction that specifically deals with policymaking and interventions, highlights that iodine deficiency elimination programs are considered in its interventional options; hence, countries with developing or no iodization programs, such as Russia, may simultaneously benefit [47]. In addition to conducting robust salt surveillance programs, one of the initially recommended steps for intervention is to reformulate non-household food to have less salt content, such as processed foods and restaurant meals, in order to offset consumer demand for salty food products. To this end, the WHO has published global sodium benchmarks to aid in the reformulation process, suggesting maximum levels of sodium content for a variety of foods [48].

Further interventional options include front-of-pack labelling (FOPL) of salt content on food products, disseminating knowledge to the public on the risks and benefits of salt intake, and carrying out salt reduction interventions at community-level settings, such as schools and workplaces. In a modelling study that examined the health impact of FOPL in Canada, it was estimated that up to 128 mg/day of sodium may be reduced following certain interventions, representing a significant reduction in sodium intake using nutritional awareness methods alone [49].

The use of potassium-based salts may also serve as a healthier alternative to sodium-based salts in terms of CVD mortality reduction. A recent study in India modelled the effect of replacing sodium salt with potassium salt on CVD events at the population level while accounting for the adverse effects of hyperkalaemia [50]. Their findings suggested that up to 14% of cardiovascular events could be prevented on an annual basis, reflecting a significant increase in the number of lives saved as a result of the intervention. The study also concluded that the benefits of cardiovascular health outweighed the risks from hyperkalaemia brought about by the intervention, even among those with chronic kidney disease. However, randomized clinical trials are needed to clearly assess the role of potassium salt substitutes as a clinical intervention for CVD mortality reduction in a population.

Effective implementation of the above WHO recommended salt reduction package will eventually result in population-level benefits of salt consumption and prevent a significant number of cardiovascular deaths, which may be largely stimulated through industry collaboration on food reformulation, as stated above. However, it should be carefully noted that interventions involving the multiple factors described above are more successful in achieving larger reductions in salt intake rather than focusing on individual strategies [51]. Such multi-component strategies are key as they better consider different population groups and sources of sodium in reducing population consumption of sodium. Thus, as countries in the EEU start to develop salt surveillance and reduction plans through this country support package, major returns on public health investments may be attained, which may further progress to achieving SDG 3.4 throughout the region.

### Impact of PRIME in similar studies

PRIME is a simple but powerful tool that can be used in designing and guiding population-level policies to control risk factors related to NCDs and its associated mortality [21]. To date, a number of notable studies have used PRIME to model interventions of salt intake on mortality: Breda et al. modelled multiple scenarios in the reduction of salt, alcohol, tobacco, and physical inactivity and found that reductions in salt intake and physical inactivity at the population-level could contribute to the largest gains in preventing NCD mortality in Turkey [52]. Vega-Solano et al. modelled salt intake interventions on the Costa Rican population to acquire specific disease mortality rates for cardiovascular related deaths [53]. A Brazilian study led by Nilson et al. utilized PRIME in conjunction with another macro-simulation model to estimate the economic impact of NCD deaths due to sodium intake, which found that over USD $700 million was lost in 2017 [54].

More recently, Vargas-Meza et al. found that cases of ischemic heart disease, hypertensive disease, and stroke were most prevented in a PRIME study examining sodium intake, similar to the findings of this study [55]. In a macrosimulation PRIME modelling study in Canada, reductions in sodium content following food reformulation were estimated to avert or delay 36.5% of individuals under 75 years of age, again reflecting the significant burden sodium salt intake has on the working population [56]. One study in Portugal also specifically analysed the impact of salt reduction in bread, a widely consumed food product, which showed that salt consumption would drop by 3.6 tons/day and result in 286 lives saved per year with this intervention alone [57].

### Study strengths and limitations

As far as we are aware, this is the first study to model the impact of salt intake interventions on NCD mortality within the EEU. Each member state’s demographic data were obtained from their respective governmental statistical bodies according to the national census. As some member states do not have national targets for salt reduction polices, this study’s findings may guide their respective policymakers in creating life-saving interventions for NCDs at the population level and highlights the importance of conducting salt surveillance studies in order to monitor their progress.

As salt intake data were not stratified by age, the corresponding findings do not account for age-related changes in salt intake. Moreover, in all surveyed countries, only those at least 18 years of age reported on salt intake. Kyrgyzstan did not have salt intake data available as a part of its STEPS surveillance study; therefore, Kazakhstan’s data were used as proxy, which may have affected the findings. In Russia, salt intake data was not stratified by gender, and Belarus’ male SD was used as a proxy as data for Russia was unavailable. This limits the accuracy of the Russian findings. NCD mortality data for heart failure (I50) were not reported for Belarus and Russia, and data for pulmonary embolism (I26) was not available for Russia, which may affect the accuracy of the model. We note that these data issues mean that our findings likely underestimate the impact of salt reduction. A further issue is that the salt surveillance data available for each country did not always correspond to the population and NCD mortality data by exact year. The largest gaps were seen in both Armenia and Belarus where there was a two-year gap; the salt data were available for 2016 but the closest available demographic and mortality data were from 2018.

The PRIME model has its own set of limitations, which are also present in this study. Specifically, as PRIME computes its risk estimates from published meta-analyses, its estimates do not correspond with population-specific risks as they are not generalizable. Additionally, due to the nature of the PRIME model, deaths due to NCD risk factors cannot be extrapolated in dynamic counterfactual scenarios. As a result, these findings cannot project preventable future deaths. To mitigate these limitations, robust salt surveillance studies using reliable means of assessment, such as the STEPS tool, can result in large enough sample sizes to more accurately reflect the NCD mortality risk for risk factors, especially salt intake.

## Conclusions

The findings of the PRIME models suggest that very large reductions in deaths can be achieved by reducing salt intake across the Eurasian Economic Union. We recommend that EEU countries should urgently implement salt reduction policies and conduct regular population-wide salt intake surveillance studies to better ascertain their level of NCD risk, particularly for cardiovascular disease, and monitor their progress in achieving the SDGs.

## Supporting information

S1 Table Population data for each EEU member state. (DOCX)

S2 Table Mortality data for Armenia (2018). (DOCX)

S3 Table Mortality data for Belarus (2018). (DOCX)

S4 Table Mortality data for Kazakhstan (2017). (DOCX)

S5 Table Mortality data for Kyrgyzstan (2016). (DOCX)

S6 Table Mortality data for Russia (2019). (DOCX)

S7 Table Total number of estimated prevented deaths by disease from a 30% reduction in salt intake. (DOCX)

S8 Table Total number of estimated prevented deaths by disease from adopting the WHO recommended salt intake. (DOCX)

S9 Table Total deaths according to age group after a 30% reduction in salt intake. (DOCX)

S10 Table Total deaths according to age group after adopting the WHO recommended salt intake. (DOCX)

S11 Table Relevant cardiovascular disease ICD-10 codes used by PRIME. (DOCX)

S1 File (ZIP)
