# Supplementary material for: Evaluating the role of salt intake in achieving WHO NCD targets in the Eurasian Economic Union: A PRIME modeling study
Source: PLoS One. 2023 Jul 21;18(7):e0289112. doi: 10.1371/journal.pone.0289112 (PMC10361522; doi:10.1371/journal.pone.0289112)
Supplement: S1 Table — (DOCX) [file pone.0289112.s001.docx]

|  | **Armenia** | | **Belarus** | | **Kazakhstan** | | **Kyrgyzstan** | | **Russia** | |
| --- | --- | --- | --- | --- | --- | --- | --- | --- | --- | --- |
|  | Male | Female | Male | Female | Male | Female | Male | Female | Male | Female |
| **15-19** | 87587 | 77649 | 232987 | 220281 | 567277 | 542223 | 260805 | 251221 | 3552070 | 3395273 |
| **20-24** | 97796 | 98087 | 263427 | 249213 | 690952 | 666951 | 290661 | 280306 | 3627097 | 3486971 |
| **25-29** | 126140 | 138537 | 365053 | 348588 | 803792 | 821277 | 290546 | 289307 | 5214083 | 5007956 |
| **30-34** | 124735 | 138007 | 397622 | 384216 | 723674 | 738732 | 231896 | 229332 | 6385234 | 6332379 |
| **35-39** | 105642 | 116735 | 349879 | 350574 | 610981 | 631231 | 187482 | 186925 | 5805924 | 5929435 |
| **40-44** | 84155 | 97212 | 318786 | 335305 | 551728 | 593373 | 162938 | 171087 | 5110238 | 5483955 |
| **45-49** | 74908 | 90396 | 303200 | 334221 | 503869 | 549310 | 151186 | 161094 | 4626578 | 5047432 |
| **50-54** | 80873 | 101053 | 308180 | 352950 | 482777 | 550722 | 138080 | 153973 | 4192011 | 4828523 |
| **55-59** | 96688 | 120578 | 335591 | 405702 | 431261 | 520895 | 114218 | 132268 | 4824036 | 5986657 |
| **60-64** | 74801 | 96903 | 263836 | 357615 | 295471 | 398685 | 71822 | 89744 | 4173981 | 5852980 |
| **65-69** | 51065 | 70992 | 195226 | 303984 | 202643 | 310749 | 44580 | 61483 | 3144349 | 5034322 |
| **70-74** | 24521 | 37238 | 267163 | 653288 | 88761 | 151689 | 18367 | 26519 | 1644926 | 2990299 |
| **75-79** | 29615 | 47872 | 267163 | 653288 | 100584 | 205010 | 22282 | 37338 | 1121922 | 2663232 |
| **80-84** | 30667 | 52623 | 267163 | 653288 | 41164 | 95125 | 10563 | 19730 | 869484 | 2387279 |
| **85+** | 30667 | 52623 | 267163 | 653288 | 22183 | 61692 | 8622 | 15794 | 490300 | 1629315 |
