# Supplementary material for: Evaluating the role of salt intake in achieving WHO NCD targets in the Eurasian Economic Union: A PRIME modeling study
Source: PLoS One. 2023 Jul 21;18(7):e0289112. doi: 10.1371/journal.pone.0289112 (PMC10361522; doi:10.1371/journal.pone.0289112)
Supplement: S2 Table — (DOCX) [file pone.0289112.s002.docx]

|  | **Males** | | | | | | | | | | | | | | | |
| --- | --- | --- | --- | --- | --- | --- | --- | --- | --- | --- | --- | --- | --- | --- | --- | --- |
| **Age** | **15-19** | **20-24** | **25-29** | **30-34** | **35-39** | **40-44** | **45-49** | **50-54** | **55-59** | **60-64** | **65-69** | **70-75** | **75-79** | **80-84** | **85+** | **Total** |
| I60-I69: Cerebrovascular diseases | 0 | 2 | 1 | 3 | 4 | 15 | 15 | 49 | 70 | 85 | 108 | 84 | 135 | 130 | 125 | 826 |
| I20-I25: Ischaemic heart diseases | 0 | 3 | 7 | 17 | 24 | 33 | 89 | 183 | 340 | 390 | 453 | 291 | 468 | 522 | 507 | 3327 |
| C00-C14: Lip, oral cavity and pharynx | 0 | 0 | 0 | 0 | 1 | 0 | 2 | 11 | 11 | 9 | 6 | 3 | 1 | 1 | 2 | 47 |
| C15: Oesophagus | 0 | 0 | 0 | 0 | 0 | 0 | 0 | 4 | 6 | 9 | 6 | 4 | 1 | 6 | 1 | 37 |
| C16: Stomach | 0 | 0 | 2 | 4 | 1 | 4 | 8 | 16 | 22 | 33 | 46 | 29 | 36 | 32 | 17 | 250 |
| C34: Bronchus and lung | 0 | 2 | 1 | 1 | 3 | 7 | 16 | 44 | 115 | 156 | 148 | 114 | 114 | 67 | 39 | 827 |
| C25: Pancreas | 0 | 0 | 0 | 1 | 4 | 2 | 8 | 16 | 30 | 27 | 25 | 19 | 28 | 18 | 16 | 194 |
| C18-20: Colorectum | 1 | 0 | 2 | 2 | 1 | 2 | 1 | 7 | 24 | 24 | 27 | 28 | 43 | 35 | 24 | 221 |
| C50: Breast | 0 | 0 | 0 | 0 | 0 | 0 | 0 | 0 | 0 | 1 | 1 | 1 | 0 | 0 | 0 | 3 |
| C54.1: Endometrium | 0 | 0 | 0 | 0 | 0 | 0 | 0 | 0 | 0 | 0 | 0 | 0 | 0 | 0 | 0 | 0 |
| C23: Gallbladder | 0 | 0 | 0 | 0 | 0 | 0 | 1 | 0 | 1 | 0 | 1 | 1 | 2 | 4 | 0 | 10 |
| C64: Kidney | 0 | 0 | 0 | 0 | 1 | 0 | 0 | 2 | 13 | 9 | 4 | 4 | 14 | 3 | 5 | 55 |
| I10-I15: Hypertensive disease | 0 | 0 | 0 | 2 | 1 | 2 | 3 | 11 | 40 | 35 | 43 | 41 | 70 | 74 | 94 | 416 |
| E11,E14: Diabetes | 0 | 0 | 0 | 0 | 1 | 3 | 4 | 6 | 18 | 32 | 24 | 16 | 24 | 23 | 9 | 160 |
| C67: Bladder cancer | 0 | 0 | 0 | 1 | 0 | 2 | 1 | 8 | 4 | 11 | 19 | 15 | 25 | 31 | 30 | 147 |
| C22: Liver cancer | 0 | 0 | 0 | 0 | 1 | 6 | 4 | 14 | 29 | 29 | 23 | 26 | 39 | 30 | 17 | 218 |
| C53: Cervix cancer | 0 | 0 | 0 | 0 | 0 | 0 | 0 | 0 | 0 | 0 | 0 | 0 | 0 | 0 | 0 | 0 |
| J40-J44: COPD | 0 | 0 | 0 | 0 | 0 | 2 | 2 | 4 | 15 | 22 | 34 | 26 | 36 | 52 | 52 | 245 |
| K70, K74: Liver disease | 0 | 0 | 1 | 2 | 4 | 14 | 24 | 36 | 30 | 35 | 36 | 15 | 28 | 32 | 27 | 284 |
| I50: Heart failure | 0 | 0 | 3 | 5 | 2 | 0 | 3 | 1 | 4 | 6 | 11 | 10 | 18 | 41 | 77 | 181 |
| I71: Aortic aneurysm | 0 | 0 | 0 | 1 | 2 | 0 | 5 | 7 | 7 | 24 | 22 | 19 | 22 | 25 | 16 | 150 |
| I26: Pulmonary embolism | 0 | 0 | 0 | 0 | 0 | 2 | 1 | 3 | 2 | 4 | 10 | 4 | 5 | 10 | 11 | 52 |
| I05-09: Rheumatic heart disease | 0 | 0 | 0 | 0 | 0 | 0 | 1 | 1 | 1 | 1 | 0 | 0 | 1 | 1 | 1 | 7 |
| N18: Chronic renal failure | 0 | 0 | 0 | 0 | 1 | 0 | 1 | 2 | 4 | 11 | 1 | 5 | 9 | 3 | 4 | 41 |
| **Total** | 1 | 7 | 17 | 39 | 51 | 94 | 189 | 425 | 786 | 953 | 1,048 | 755 | 1,119 | 1,140 | 1,074 | 7,698 |
|  |  |  |  |  |  |  |  |  |  |  |  |  |  |  |  |  |
|  | **Females** | | | | | | | | | | | | | | | |
| **Age** | **15-19** | **20-24** | **25-29** | **30-34** | **35-39** | **40-44** | **45-49** | **50-54** | **55-59** | **60-64** | **65-69** | **70-75** | **75-79** | **80-84** | **85+** | **Total** |
| I60-I69: Cerebrovascular diseases | 0 | 0 | 0 | 2 | 3 | 3 | 8 | 8 | 35 | 58 | 63 | 75 | 191 | 276 | 299 | 1021 |
| I20-I25: Ischaemic heart diseases | 0 | 0 | 4 | 3 | 5 | 10 | 22 | 40 | 100 | 150 | 230 | 223 | 520 | 733 | 967 | 3007 |
| C00-C14: Lip, oral cavity and pharynx | 0 | 0 | 0 | 0 | 1 | 1 | 0 | 0 | 0 | 3 | 2 | 2 | 4 | 0 | 2 | 15 |
| C15: Oesophagus | 0 | 0 | 0 | 0 | 0 | 0 | 0 | 0 | 0 | 3 | 3 | 1 | 1 | 1 | 3 | 12 |
| C16: Stomach | 1 | 0 | 0 | 1 | 3 | 5 | 4 | 7 | 10 | 18 | 27 | 11 | 28 | 33 | 20 | 168 |
| C34: Bronchus and lung | 0 | 1 | 0 | 1 | 3 | 5 | 2 | 6 | 18 | 22 | 23 | 14 | 32 | 30 | 29 | 186 |
| C25: Pancreas | 0 | 0 | 0 | 2 | 1 | 0 | 4 | 2 | 21 | 21 | 22 | 25 | 32 | 33 | 20 | 183 |
| C18-20: Colorectum | 0 | 0 | 0 | 2 | 0 | 2 | 4 | 11 | 20 | 28 | 26 | 32 | 59 | 40 | 36 | 260 |
| C50: Breast | 0 | 0 | 0 | 6 | 4 | 10 | 22 | 41 | 50 | 58 | 60 | 36 | 48 | 39 | 29 | 403 |
| C54.1: Endometrium | 0 | 0 | 0 | 0 | 1 | 2 | 4 | 6 | 9 | 12 | 14 | 12 | 13 | 12 | 5 | 90 |
| C23: Gallbladder | 0 | 0 | 0 | 0 | 0 | 0 | 0 | 1 | 4 | 4 | 3 | 4 | 5 | 5 | 4 | 30 |
| C64: Kidney | 0 | 0 | 0 | 0 | 0 | 1 | 0 | 0 | 4 | 5 | 1 | 4 | 5 | 3 | 7 | 30 |
| I10-I15: Hypertensive disease | 0 | 0 | 0 | 0 | 0 | 0 | 1 | 9 | 16 | 31 | 42 | 50 | 94 | 159 | 210 | 612 |
| E11,E14: Diabetes | 0 | 0 | 0 | 1 | 1 | 1 | 1 | 3 | 22 | 27 | 41 | 37 | 59 | 44 | 26 | 263 |
| C67: Bladder cancer | 0 | 0 | 0 | 0 | 0 | 0 | 3 | 0 | 2 | 0 | 4 | 4 | 3 | 5 | 6 | 27 |
| C22: Liver cancer | 0 | 0 | 0 | 2 | 1 | 3 | 3 | 8 | 19 | 24 | 20 | 23 | 26 | 28 | 27 | 184 |
| C53: Cervix cancer | 0 | 0 | 0 | 2 | 3 | 7 | 2 | 6 | 11 | 9 | 4 | 2 | 8 | 1 | 3 | 58 |
| J40-J44: COPD | 0 | 0 | 0 | 0 | 0 | 0 | 1 | 1 | 3 | 3 | 6 | 6 | 27 | 30 | 57 | 134 |
| K70, K74: Liver disease | 0 | 1 | 0 | 1 | 2 | 0 | 1 | 4 | 9 | 23 | 30 | 13 | 33 | 43 | 68 | 228 |
| I50: Heart failure | 0 | 0 | 2 | 0 | 1 | 1 | 2 | 2 | 3 | 6 | 8 | 10 | 40 | 68 | 141 | 284 |
| I71: Aortic aneurysm | 0 | 0 | 1 | 0 | 0 | 0 | 2 | 3 | 8 | 4 | 10 | 12 | 13 | 22 | 20 | 95 |
| I26: Pulmonary embolism | 0 | 0 | 0 | 0 | 0 | 0 | 1 | 2 | 0 | 4 | 2 | 4 | 14 | 16 | 27 | 70 |
| I05-09: Rheumatic heart disease | 0 | 0 | 1 | 0 | 0 | 1 | 1 | 1 | 1 | 5 | 4 | 2 | 2 | 1 | 2 | 21 |
| N18: Chronic renal failure | 0 | 1 | 0 | 0 | 2 | 2 | 0 | 6 | 1 | 6 | 3 | 5 | 6 | 11 | 4 | 47 |
| **Total** | 1 | 3 | 8 | 23 | 31 | 54 | 88 | 167 | 366 | 524 | 648 | 607 | 1,263 | 1,633 | 2,012 | 7,428 |
|  |  |  |  |  |  |  |  |  |  |  |  |  |  |  |  |  |
