# Supplementary material for: Evaluating the role of salt intake in achieving WHO NCD targets in the Eurasian Economic Union: A PRIME modeling study
Source: PLoS One. 2023 Jul 21;18(7):e0289112. doi: 10.1371/journal.pone.0289112 (PMC10361522; doi:10.1371/journal.pone.0289112)
Supplement: S3 Table — (DOCX) [file pone.0289112.s003.docx]

|  | **Males** | | | | | | | | | | | | | | | |
| --- | --- | --- | --- | --- | --- | --- | --- | --- | --- | --- | --- | --- | --- | --- | --- | --- |
| **Age** | **15-19** | **20-24** | **25-29** | **30-34** | **35-39** | **40-44** | **45-49** | **50-54** | **55-59** | **60-64** | **65-69** | **70-75** | **75-79** | **80-84** | **85+** | **Total** |
| I60-I69: Cerebrovascular diseases | 0 | 1 | 4 | 18 | 47 | 86 | 151 | 253 | 530 | 771 | 884 | 713 | 824 | 744 | 651 | 5677 |
| I20-I25: Ischaemic heart diseases | 0 | 6 | 16 | 73 | 138 | 288 | 616 | 1042 | 2004 | 3085 | 3545 | 2876 | 3411 | 3407 | 3139 | 23646 |
| C00-C14: Lip, oral cavity and pharynx | 0 | 0 | 0 | 1 | 14 | 23 | 71 | 129 | 189 | 175 | 128 | 68 | 25 | 11 | 6 | 840 |
| C15: Oesophagus | 0 | 0 | 0 | 1 | 3 | 7 | 29 | 42 | 77 | 89 | 69 | 41 | 18 | 14 | 5 | 395 |
| C16: Stomach | 0 | 0 | 4 | 9 | 8 | 32 | 35 | 67 | 164 | 190 | 190 | 121 | 133 | 64 | 47 | 1064 |
| C34: Bronchus and lung | 0 | 0 | 0 | 1 | 6 | 28 | 62 | 198 | 467 | 609 | 540 | 338 | 284 | 139 | 59 | 2731 |
| C25: Pancreas | 0 | 0 | 0 | 6 | 5 | 11 | 25 | 46 | 109 | 112 | 115 | 58 | 42 | 32 | 22 | 583 |
| C18-20: Colorectum | 0 | 0 | 0 | 3 | 11 | 11 | 20 | 58 | 132 | 202 | 217 | 160 | 184 | 118 | 80 | 1196 |
| C50: Breast | 0 | 0 | 0 | 0 | 0 | 0 | 0 | 1 | 2 | 2 | 1 | 0 | 0 | 0 | 0 | 6 |
| C54.1: Endometrium | 0 | 0 | 0 | 0 | 0 | 0 | 0 | 0 | 0 | 0 | 0 | 0 | 0 | 0 | 0 | 0 |
| C23: Gallbladder | 0 | 0 | 0 | 0 | 0 | 0 | 1 | 0 | 4 | 1 | 3 | 3 | 2 | 1 | 0 | 15 |
| C64: Kidney | 0 | 1 | 0 | 1 | 1 | 5 | 13 | 30 | 79 | 67 | 63 | 47 | 35 | 16 | 11 | 369 |
| I10-I15: Hypertensive disease | 0 | 0 | 0 | 2 | 1 | 5 | 3 | 8 | 13 | 17 | 9 | 10 | 6 | 1 | 3 | 78 |
| E11,E14: Diabetes | 0 | 0 | 0 | 0 | 2 | 0 | 3 | 1 | 15 | 11 | 23 | 10 | 8 | 4 | 1 | 78 |
| C67: Bladder cancer | 0 | 0 | 0 | 0 | 0 | 0 | 7 | 6 | 37 | 49 | 45 | 39 | 53 | 46 | 20 | 302 |
| C22: Liver cancer | 0 | 0 | 1 | 0 | 0 | 4 | 15 | 34 | 34 | 71 | 65 | 36 | 31 | 13 | 4 | 308 |
| C53: Cervix cancer | 0 | 0 | 0 | 0 | 0 | 0 | 0 | 0 | 0 | 0 | 0 | 0 | 0 | 0 | 0 | 0 |
| J40-J44: COPD | 0 | 0 | 0 | 3 | 3 | 4 | 13 | 34 | 72 | 121 | 148 | 131 | 145 | 119 | 80 | 873 |
| K70, K74: Liver disease | 0 | 0 | 6 | 25 | 61 | 95 | 132 | 125 | 145 | 182 | 125 | 52 | 35 | 16 | 6 | 1005 |
| I50: Heart failure | N/A | N/A | N/A | N/A | N/A | N/A | N/A | N/A | N/A | N/A | N/A | N/A | N/A | N/A | N/A | 0 |
| I71: Aortic aneurysm | 0 | 0 | 1 | 3 | 5 | 7 | 18 | 27 | 55 | 66 | 68 | 50 | 48 | 27 | 11 | 386 |
| I26: Pulmonary embolism | 0 | 0 | 0 | 0 | 0 | 0 | 2 | 1 | 1 | 4 | 2 | 1 | 1 | 0 | 0 | 12 |
| I05-09: Rheumatic heart disease | 0 | 0 | 0 | 1 | 0 | 1 | 4 | 13 | 13 | 14 | 8 | 6 | 2 | 2 | 0 | 64 |
| N18: Chronic renal failure | 0 | 0 | 0 | 0 | 0 | 0 | 2 | 4 | 4 | 3 | 4 | 3 | 4 | 1 | 1 | 26 |
| **Total** | 0 | 8 | 32 | 147 | 305 | 607 | 1,222 | 2,119 | 4,146 | 5,841 | 6,252 | 4,763 | 5,291 | 4,775 | 4,146 | 39,654 |
|  |  |  |  |  |  |  |  |  |  |  |  |  |  |  |  |  |
|  | **Females** | | | | | | | | | | | | | | | |
| **Age** | **15-19** | **20-24** | **25-29** | **30-34** | **35-39** | **40-44** | **45-49** | **50-54** | **55-59** | **60-64** | **65-69** | **70-75** | **75-79** | **80-84** | **85+** | **Total** |
| I60-I69: Cerebrovascular diseases | 1 | 1 | 3 | 8 | 21 | 40 | 57 | 126 | 263 | 376 | 577 | 666 | 1327 | 1794 | 2323 | 7583 |
| I20-I25: Ischaemic heart diseases | 0 | 0 | 2 | 12 | 18 | 65 | 109 | 195 | 544 | 1044 | 1710 | 2098 | 4584 | 6113 | 9542 | 26036 |
| C00-C14: Lip, oral cavity and pharynx | 0 | 0 | 0 | 1 | 4 | 4 | 4 | 12 | 11 | 16 | 17 | 10 | 13 | 15 | 12 | 119 |
| C15: Oesophagus | 0 | 0 | 0 | 0 | 0 | 1 | 2 | 5 | 6 | 1 | 6 | 6 | 7 | 3 | 3 | 40 |
| C16: Stomach | 1 | 0 | 4 | 7 | 7 | 19 | 26 | 25 | 73 | 68 | 96 | 68 | 104 | 99 | 82 | 679 |
| C34: Bronchus and lung | 0 | 0 | 1 | 2 | 4 | 5 | 9 | 20 | 30 | 64 | 69 | 46 | 60 | 43 | 28 | 381 |
| C25: Pancreas | 0 | 1 | 1 | 0 | 4 | 3 | 10 | 21 | 32 | 46 | 74 | 61 | 74 | 64 | 42 | 433 |
| C18-20: Colorectum | 0 | 1 | 1 | 2 | 6 | 15 | 19 | 49 | 105 | 132 | 187 | 136 | 204 | 190 | 135 | 1182 |
| C50: Breast | 0 | 0 | 2 | 6 | 24 | 35 | 75 | 93 | 130 | 177 | 192 | 114 | 112 | 106 | 72 | 1138 |
| C54.1: Endometrium | 0 | 0 | 0 | 0 | 1 | 4 | 7 | 15 | 26 | 67 | 82 | 54 | 48 | 56 | 21 | 381 |
| C23: Gallbladder | 0 | 0 | 0 | 0 | 0 | 1 | 3 | 2 | 4 | 6 | 15 | 7 | 11 | 11 | 12 | 72 |
| C64: Kidney | 0 | 0 | 1 | 1 | 0 | 1 | 1 | 6 | 12 | 21 | 41 | 22 | 32 | 32 | 8 | 178 |
| I10-I15: Hypertensive disease | 0 | 0 | 0 | 0 | 0 | 1 | 4 | 7 | 7 | 8 | 7 | 10 | 6 | 11 | 5 | 66 |
| E11,E14: Diabetes | 0 | 0 | 0 | 0 | 0 | 1 | 1 | 4 | 10 | 11 | 21 | 18 | 23 | 11 | 10 | 110 |
| C67: Bladder cancer | 0 | 0 | 0 | 0 | 0 | 0 | 2 | 0 | 2 | 1 | 11 | 6 | 3 | 15 | 14 | 54 |
| C22: Liver cancer | 0 | 0 | 2 | 2 | 3 | 1 | 7 | 5 | 11 | 11 | 20 | 14 | 31 | 18 | 17 | 142 |
| C53: Cervix cancer | 0 | 0 | 2 | 5 | 19 | 23 | 35 | 42 | 41 | 42 | 29 | 20 | 16 | 21 | 11 | 306 |
| J40-J44: COPD | 0 | 0 | 0 | 0 | 0 | 1 | 0 | 7 | 8 | 16 | 20 | 25 | 36 | 66 | 54 | 233 |
| K70, K74: Liver disease | 0 | 0 | 7 | 20 | 27 | 48 | 63 | 80 | 166 | 111 | 85 | 43 | 34 | 17 | 8 | 709 |
| I50: Heart failure | N/A | N/A | N/A | N/A | N/A | N/A | N/A | N/A | N/A | N/A | N/A | N/A | N/A | N/A | N/A | 0 |
| I71: Aortic aneurysm | 0 | 0 | 1 | 1 | 6 | 5 | 10 | 11 | 18 | 13 | 8 | 14 | 18 | 26 | 30 | 161 |
| I26: Pulmonary embolism | 0 | 0 | 0 | 1 | 0 | 2 | 2 | 0 | 1 | 2 | 2 | 0 | 4 | 2 | 1 | 17 |
| I05-09: Rheumatic heart disease | 0 | 0 | 0 | 0 | 1 | 1 | 0 | 4 | 16 | 21 | 26 | 16 | 16 | 14 | 7 | 122 |
| N18: Chronic renal failure | 0 | 0 | 0 | 0 | 0 | 1 | 0 | 1 | 0 | 0 | 5 | 1 | 5 | 5 | 1 | 19 |
| **Total** | 2 | 3 | 27 | 68 | 145 | 277 | 446 | 730 | 1,516 | 2,254 | 3,300 | 3,455 | 6,768 | 8,732 | 12,438 | 40,161 |
|  |  |  |  |  |  |  |  |  |  |  |  |  |  |  |  |  |
