# Supplementary material for: Evaluating the role of salt intake in achieving WHO NCD targets in the Eurasian Economic Union: A PRIME modeling study
Source: PLoS One. 2023 Jul 21;18(7):e0289112. doi: 10.1371/journal.pone.0289112 (PMC10361522; doi:10.1371/journal.pone.0289112)
Supplement: S4 Table — (DOCX) [file pone.0289112.s004.docx]

|  | **Males** | | | | | | | | | | | | | | | |
| --- | --- | --- | --- | --- | --- | --- | --- | --- | --- | --- | --- | --- | --- | --- | --- | --- |
| **Age** | **15-19** | **20-24** | **25-29** | **30-34** | **35-39** | **40-44** | **45-49** | **50-54** | **55-59** | **60-64** | **65-69** | **70-75** | **75-79** | **80-84** | **85+** | **Total** |
| I60-I69: Cerebrovascular diseases | 9 | 18 | 24 | 56 | 99 | 181 | 314 | 481 | 747 | 782 | 880 | 577 | 864 | 495 | 332 | 5859 |
| I20-I25: Ischaemic heart diseases | 0 | 3 | 22 | 38 | 98 | 196 | 372 | 644 | 985 | 1037 | 1081 | 621 | 860 | 480 | 334 | 6771 |
| C00-C14: Lip, oral cavity and pharynx | 1 | 2 | 0 | 3 | 7 | 7 | 21 | 36 | 65 | 63 | 59 | 26 | 19 | 11 | 2 | 322 |
| C15: Oesophagus | 0 | 1 | 0 | 1 | 1 | 6 | 21 | 19 | 73 | 89 | 88 | 44 | 55 | 18 | 13 | 429 |
| C16: Stomach | 0 | 1 | 5 | 11 | 27 | 27 | 50 | 97 | 180 | 199 | 200 | 106 | 120 | 55 | 16 | 1094 |
| C34: Bronchus and lung | 0 | 0 | 3 | 8 | 8 | 32 | 65 | 178 | 359 | 443 | 442 | 187 | 203 | 63 | 15 | 2006 |
| C25: Pancreas | 0 | 0 | 2 | 3 | 5 | 11 | 24 | 49 | 77 | 89 | 80 | 39 | 36 | 16 | 2 | 433 |
| C18-20: Colorectum | 0 | 2 | 4 | 4 | 8 | 17 | 33 | 63 | 92 | 128 | 149 | 84 | 99 | 48 | 17 | 748 |
| C50: Breast | 0 | 0 | 0 | 0 | 0 | 0 | 0 | 0 | 0 | 0 | 0 | 1 | 1 | 0 | 0 | 2 |
| C54.1: Endometrium | 0 | 0 | 0 | 0 | 0 | 0 | 0 | 0 | 0 | 0 | 0 | 0 | 0 | 0 | 0 | 0 |
| C23: Gallbladder | 0 | 0 | 0 | 0 | 0 | 0 | 1 | 2 | 4 | 4 | 2 | 2 | 0 | 0 | 0 | 15 |
| C64: Kidney | 0 | 0 | 2 | 1 | 4 | 11 | 11 | 35 | 44 | 54 | 36 | 19 | 16 | 8 | 4 | 245 |
| I10-I15: Hypertensive disease | 1 | 0 | 1 | 2 | 10 | 13 | 21 | 43 | 52 | 77 | 80 | 34 | 51 | 38 | 24 | 447 |
| E11,E14: Diabetes | 0 | 2 | 3 | 1 | 1 | 8 | 33 | 61 | 124 | 192 | 208 | 148 | 236 | 126 | 61 | 1204 |
| C67: Bladder cancer | 0 | 0 | 0 | 0 | 1 | 7 | 8 | 11 | 21 | 39 | 33 | 21 | 33 | 25 | 15 | 214 |
| C22: Liver cancer | 0 | 0 | 2 | 3 | 1 | 7 | 15 | 34 | 62 | 75 | 68 | 35 | 47 | 15 | 9 | 373 |
| C53: Cervix cancer | 0 | 0 | 0 | 0 | 0 | 0 | 0 | 0 | 0 | 0 | 0 | 0 | 0 | 0 | 0 | 0 |
| J40-J44: COPD | 4 | 5 | 11 | 22 | 49 | 65 | 126 | 232 | 481 | 785 | 1,022 | 868 | 1,523 | 1,014 | 618 | 6825 |
| K70, K74: Liver disease | 1 | 4 | 30 | 77 | 193 | 283 | 358 | 447 | 573 | 613 | 541 | 305 | 374 | 188 | 59 | 4046 |
| I50: Heart failure | 2 | 2 | 8 | 7 | 18 | 16 | 32 | 46 | 77 | 73 | 93 | 73 | 113 | 47 | 33 | 640 |
| I71: Aortic aneurysm | 0 | 0 | 2 | 3 | 6 | 6 | 11 | 20 | 35 | 34 | 30 | 17 | 26 | 7 | 6 | 203 |
| I26: Pulmonary embolism | 0 | 0 | 6 | 3 | 9 | 18 | 17 | 25 | 32 | 37 | 34 | 24 | 20 | 4 | 4 | 233 |
| I05-09: Rheumatic heart disease | 0 | 0 | 1 | 1 | 2 | 10 | 7 | 16 | 23 | 23 | 10 | 9 | 6 | 1 | 1 | 110 |
| N18: Chronic renal failure | 1 | 4 | 6 | 10 | 18 | 17 | 26 | 44 | 56 | 70 | 95 | 61 | 98 | 62 | 28 | 596 |
| **Total** | 19 | 44 | 132 | 254 | 565 | 938 | 1,566 | 2,583 | 4,162 | 4,906 | 5,231 | 3,301 | 4,800 | 2,721 | 1,593 | 32,815 |
|  |  |  |  |  |  |  |  |  |  |  |  |  |  |  |  |  |
|  | **Females** | | | | | | | | | | | | | | | |
| **Age** | **15-19** | **20-24** | **25-29** | **30-34** | **35-39** | **40-44** | **45-49** | **50-54** | **55-59** | **60-64** | **65-69** | **70-75** | **75-79** | **80-84** | **85+** | **Total** |
| I60-I69: Cerebrovascular diseases | 2 | 3 | 7 | 28 | 58 | 90 | 156 | 255 | 386 | 476 | 675 | 505 | 1228 | 995 | 1105 | 5969 |
| I20-I25: Ischaemic heart diseases | 0 | 2 | 5 | 15 | 17 | 43 | 79 | 145 | 250 | 376 | 558 | 401 | 1054 | 843 | 905 | 4693 |
| C00-C14: Lip, oral cavity and pharynx | 0 | 0 | 4 | 2 | 2 | 12 | 17 | 15 | 29 | 22 | 24 | 10 | 11 | 10 | 8 | 166 |
| C15: Oesophagus | 0 | 0 | 4 | 2 | 1 | 6 | 12 | 23 | 31 | 39 | 57 | 37 | 69 | 27 | 13 | 321 |
| C16: Stomach | 0 | 1 | 8 | 16 | 9 | 17 | 29 | 46 | 63 | 77 | 118 | 68 | 107 | 48 | 14 | 621 |
| C34: Bronchus and lung | 0 | 0 | 1 | 5 | 11 | 14 | 21 | 31 | 57 | 81 | 64 | 62 | 61 | 33 | 13 | 454 |
| C25: Pancreas | 0 | 0 | 2 | 2 | 5 | 8 | 11 | 24 | 62 | 58 | 76 | 39 | 63 | 26 | 9 | 385 |
| C18-20: Colorectum | 0 | 3 | 5 | 8 | 16 | 18 | 30 | 42 | 68 | 113 | 123 | 76 | 127 | 66 | 32 | 727 |
| C50: Breast | 0 | 2 | 4 | 18 | 47 | 62 | 128 | 145 | 200 | 172 | 173 | 85 | 110 | 40 | 28 | 1214 |
| C54.1: Endometrium | 0 | 0 | 1 | 2 | 1 | 2 | 6 | 19 | 37 | 46 | 44 | 32 | 22 | 15 | 4 | 231 |
| C23: Gallbladder | 0 | 0 | 0 | 0 | 1 | 1 | 2 | 5 | 8 | 5 | 8 | 3 | 12 | 4 | 1 | 50 |
| C64: Kidney | 0 | 0 | 0 | 1 | 2 | 4 | 4 | 6 | 10 | 17 | 17 | 11 | 23 | 9 | 2 | 106 |
| I10-I15: Hypertensive disease | 0 | 0 | 0 | 0 | 5 | 6 | 12 | 13 | 38 | 37 | 53 | 41 | 92 | 53 | 59 | 409 |
| E11,E14: Diabetes | 0 | 1 | 4 | 3 | 4 | 6 | 15 | 37 | 127 | 224 | 353 | 294 | 546 | 389 | 276 | 2279 |
| C67: Bladder cancer | 0 | 0 | 0 | 0 | 1 | 0 | 1 | 1 | 3 | 4 | 3 | 4 | 14 | 7 | 4 | 42 |
| C22: Liver cancer | 0 | 1 | 1 | 3 | 5 | 5 | 16 | 19 | 30 | 45 | 55 | 33 | 49 | 24 | 14 | 300 |
| C53: Cervix cancer | 0 | 1 | 13 | 22 | 52 | 75 | 92 | 92 | 67 | 65 | 48 | 36 | 30 | 14 | 3 | 610 |
| J40-J44: COPD | 2 | 2 | 8 | 7 | 17 | 20 | 54 | 96 | 169 | 266 | 441 | 438 | 1,237 | 1,064 | 1,196 | 5017 |
| K70, K74: Liver disease | 0 | 4 | 17 | 41 | 84 | 149 | 163 | 221 | 353 | 405 | 404 | 286 | 515 | 299 | 234 | 3175 |
| I50: Heart failure | 2 | 3 | 2 | 3 | 6 | 10 | 10 | 15 | 36 | 51 | 69 | 64 | 107 | 78 | 87 | 543 |
| I71: Aortic aneurysm | 0 | 0 | 0 | 1 | 3 | 3 | 3 | 10 | 16 | 11 | 21 | 9 | 12 | 5 | 2 | 96 |
| I26: Pulmonary embolism | 0 | 0 | 0 | 4 | 4 | 8 | 10 | 11 | 19 | 24 | 29 | 14 | 27 | 14 | 10 | 174 |
| I05-09: Rheumatic heart disease | 2 | 0 | 3 | 3 | 4 | 4 | 8 | 11 | 12 | 26 | 21 | 11 | 26 | 9 | 0 | 140 |
| N18: Chronic renal failure | 0 | 0 | 4 | 5 | 9 | 12 | 23 | 28 | 49 | 54 | 72 | 51 | 93 | 79 | 64 | 543 |
| **Total** | 8 | 23 | 93 | 191 | 364 | 575 | 902 | 1,310 | 2,120 | 2,694 | 3,506 | 2,610 | 5,635 | 4,151 | 4,083 | 28,265 |
|  |  |  |  |  |  |  |  |  |  |  |  |  |  |  |  |  |
