# Supplementary material for: Evaluating the role of salt intake in achieving WHO NCD targets in the Eurasian Economic Union: A PRIME modeling study
Source: PLoS One. 2023 Jul 21;18(7):e0289112. doi: 10.1371/journal.pone.0289112 (PMC10361522; doi:10.1371/journal.pone.0289112)
Supplement: S5 Table — (DOCX) [file pone.0289112.s005.docx]

|  | **Males** | | | | | | | | | | | | | | | |
| --- | --- | --- | --- | --- | --- | --- | --- | --- | --- | --- | --- | --- | --- | --- | --- | --- |
| **Age** | **15-19** | **20-24** | **25-29** | **30-34** | **35-39** | **40-44** | **45-49** | **50-54** | **55-59** | **60-64** | **65-69** | **70-75** | **75-79** | **80-84** | **85+** | **Total** |
| I60-I69: Cerebrovascular diseases | 0 | 2 | 9 | 13 | 30 | 56 | 91 | 173 | 262 | 324 | 309 | 147 | 321 | 233 | 192 | 2162 |
| I20-I25: Ischaemic heart diseases | 0 | 0 | 6 | 20 | 40 | 75 | 166 | 234 | 420 | 551 | 608 | 420 | 963 | 882 | 945 | 5330 |
| C00-C14: Lip, oral cavity and pharynx | 0 | 1 | 2 | 0 | 1 | 4 | 6 | 3 | 14 | 21 | 16 | 3 | 4 | 7 | 2 | 84 |
| C15: Oesophagus | 0 | 0 | 1 | 0 | 0 | 0 | 1 | 5 | 13 | 23 | 14 | 10 | 19 | 11 | 7 | 104 |
| C16: Stomach | 0 | 0 | 0 | 6 | 12 | 15 | 38 | 52 | 75 | 86 | 72 | 42 | 57 | 29 | 7 | 491 |
| C34: Bronchus and lung | 0 | 0 | 1 | 5 | 2 | 2 | 13 | 31 | 66 | 78 | 69 | 33 | 41 | 16 | 8 | 365 |
| C25: Pancreas | 0 | 0 | 0 | 1 | 1 | 5 | 0 | 12 | 12 | 18 | 22 | 9 | 17 | 6 | 1 | 104 |
| C18-20: Colorectum | 1 | 0 | 2 | 3 | 3 | 5 | 2 | 6 | 13 | 13 | 18 | 4 | 21 | 9 | 1 | 101 |
| C50: Breast | 0 | 0 | 0 | 0 | 0 | 0 | 0 | 0 | 0 | 0 | 0 | 1 | 0 | 0 | 0 | 1 |
| C54.1: Endometrium | 0 | 0 | 0 | 0 | 0 | 0 | 0 | 0 | 0 | 0 | 0 | 0 | 0 | 0 | 0 | 0 |
| C23: Gallbladder | 0 | 0 | 0 | 0 | 1 | 0 | 1 | 0 | 5 | 1 | 3 | 1 | 0 | 0 | 0 | 12 |
| C64: Kidney | 0 | 0 | 0 | 1 | 0 | 0 | 5 | 2 | 10 | 6 | 6 | 3 | 2 | 0 | 1 | 36 |
| I10-I15: Hypertensive disease | 0 | 0 | 0 | 0 | 0 | 4 | 2 | 14 | 23 | 28 | 30 | 17 | 41 | 19 | 32 | 210 |
| E11,E14: Diabetes | 1 | 0 | 1 | 3 | 1 | 4 | 5 | 12 | 20 | 16 | 16 | 9 | 6 | 2 | 0 | 96 |
| C67: Bladder cancer | 0 | 0 | 0 | 0 | 0 | 0 | 1 | 1 | 3 | 2 | 8 | 0 | 5 | 2 | 1 | 23 |
| C22: Liver cancer | 0 | 1 | 1 | 3 | 5 | 4 | 11 | 19 | 33 | 37 | 32 | 18 | 26 | 11 | 2 | 203 |
| C53: Cervix cancer | 0 | 0 | 0 | 0 | 0 | 0 | 0 | 0 | 0 | 0 | 0 | 0 | 0 | 0 | 0 | 0 |
| J40-J44: COPD | 1 | 0 | 3 | 4 | 7 | 7 | 10 | 24 | 47 | 63 | 71 | 66 | 127 | 97 | 125 | 652 |
| K70, K74: Liver disease | 3 | 6 | 25 | 54 | 77 | 128 | 151 | 155 | 190 | 114 | 86 | 38 | 45 | 25 | 13 | 1110 |
| I50: Heart failure | 0 | 0 | 0 | 0 | 1 | 1 | 3 | 5 | 7 | 9 | 7 | 3 | 6 | 2 | 9 | 53 |
| I71: Aortic aneurysm | 0 | 0 | 0 | 0 | 0 | 0 | 1 | 0 | 2 | 3 | 1 | 2 | 1 | 0 | 0 | 10 |
| I26: Pulmonary embolism | 0 | 0 | 1 | 1 | 2 | 2 | 6 | 7 | 14 | 10 | 11 | 3 | 2 | 4 | 1 | 64 |
| I05-09: Rheumatic heart disease | 3 | 0 | 2 | 3 | 0 | 6 | 10 | 8 | 9 | 7 | 6 | 1 | 2 | 0 | 0 | 57 |
| N18: Chronic renal failure | 0 | 4 | 3 | 3 | 5 | 5 | 6 | 6 | 5 | 7 | 2 | 1 | 1 | 0 | 0 | 48 |
| **Total** | 9 | 14 | 57 | 120 | 188 | 323 | 529 | 769 | 1,243 | 1,417 | 1,407 | 831 | 1,707 | 1,355 | 1,347 | 11,316 |
|  |  |  |  |  |  |  |  |  |  |  |  |  |  |  |  |  |
|  | **Females** | | | | | | | | | | | | | | | |
| **Age** | **15-19** | **20-24** | **25-29** | **30-34** | **35-39** | **40-44** | **45-49** | **50-54** | **55-59** | **60-64** | **65-69** | **70-75** | **75-79** | **80-84** | **85+** | **Total** |
| I60-I69: Cerebrovascular diseases | 4 | 3 | 4 | 6 | 9 | 37 | 44 | 86 | 141 | 206 | 216 | 144 | 365 | 319 | 397 | 1981 |
| I20-I25: Ischaemic heart diseases | 2 | 1 | 0 | 4 | 3 | 24 | 50 | 87 | 206 | 289 | 449 | 423 | 1083 | 1219 | 1946 | 5786 |
| C00-C14: Lip, oral cavity and pharynx | 0 | 1 | 2 | 1 | 3 | 6 | 0 | 3 | 7 | 5 | 3 | 1 | 3 | 4 | 4 | 43 |
| C15: Oesophagus | 0 | 0 | 0 | 0 | 2 | 0 | 3 | 5 | 8 | 6 | 6 | 6 | 9 | 8 | 3 | 56 |
| C16: Stomach | 0 | 1 | 2 | 4 | 5 | 11 | 10 | 28 | 17 | 25 | 37 | 12 | 27 | 17 | 1 | 197 |
| C34: Bronchus and lung | 0 | 1 | 0 | 0 | 1 | 2 | 9 | 11 | 16 | 27 | 27 | 13 | 18 | 9 | 4 | 138 |
| C25: Pancreas | 0 | 1 | 2 | 0 | 0 | 1 | 8 | 7 | 14 | 18 | 15 | 7 | 12 | 11 | 8 | 104 |
| C18-20: Colorectum | 0 | 0 | 1 | 3 | 2 | 1 | 3 | 7 | 18 | 15 | 15 | 13 | 14 | 12 | 5 | 109 |
| C50: Breast | 0 | 0 | 1 | 5 | 7 | 9 | 24 | 25 | 37 | 29 | 23 | 14 | 23 | 16 | 10 | 223 |
| C54.1: Endometrium | 0 | 0 | 0 | 0 | 1 | 2 | 6 | 6 | 10 | 14 | 13 | 5 | 5 | 4 | 0 | 66 |
| C23: Gallbladder | 0 | 0 | 0 | 0 | 0 | 1 | 0 | 1 | 0 | 7 | 3 | 1 | 1 | 1 | 1 | 16 |
| C64: Kidney | 0 | 0 | 0 | 0 | 0 | 2 | 0 | 2 | 5 | 7 | 3 | 3 | 1 | 1 | 3 | 27 |
| I10-I15: Hypertensive disease | 1 | 0 | 0 | 0 | 4 | 0 | 2 | 6 | 11 | 21 | 18 | 24 | 35 | 28 | 42 | 192 |
| E11,E14: Diabetes | 0 | 0 | 2 | 1 | 1 | 4 | 5 | 14 | 21 | 25 | 31 | 14 | 21 | 11 | 1 | 151 |
| C67: Bladder cancer | 0 | 0 | 0 | 0 | 0 | 0 | 0 | 1 | 1 | 0 | 0 | 0 | 3 | 5 | 2 | 12 |
| C22: Liver cancer | 0 | 1 | 2 | 0 | 4 | 3 | 4 | 9 | 14 | 19 | 17 | 16 | 23 | 14 | 8 | 134 |
| C53: Cervix cancer | 0 | 0 | 1 | 7 | 11 | 27 | 38 | 25 | 33 | 31 | 15 | 10 | 12 | 8 | 4 | 222 |
| J40-J44: COPD | 0 | 0 | 1 | 1 | 1 | 2 | 4 | 7 | 22 | 37 | 42 | 30 | 83 | 112 | 160 | 502 |
| K70, K74: Liver disease | 2 | 3 | 6 | 14 | 26 | 54 | 51 | 55 | 87 | 72 | 65 | 34 | 52 | 20 | 25 | 566 |
| I50: Heart failure | 0 | 0 | 0 | 1 | 0 | 1 | 2 | 4 | 1 | 3 | 1 | 2 | 2 | 5 | 16 | 38 |
| I71: Aortic aneurysm | 0 | 0 | 0 | 0 | 0 | 0 | 0 | 0 | 1 | 1 | 0 | 0 | 2 | 0 | 2 | 6 |
| I26: Pulmonary embolism | 0 | 1 | 0 | 0 | 5 | 2 | 2 | 3 | 4 | 7 | 5 | 2 | 3 | 2 | 3 | 39 |
| I05-09: Rheumatic heart disease | 0 | 2 | 3 | 2 | 3 | 6 | 11 | 13 | 8 | 17 | 11 | 1 | 5 | 1 | 0 | 83 |
| N18: Chronic renal failure | 1 | 0 | 2 | 3 | 0 | 2 | 1 | 4 | 2 | 4 | 4 | 3 | 3 | 1 | 1 | 31 |
| **Total** | 10 | 15 | 29 | 52 | 88 | 197 | 277 | 409 | 684 | 885 | 1,019 | 778 | 1,805 | 1,828 | 2,646 | 10,722 |
|  |  |  |  |  |  |  |  |  |  |  |  |  |  |  |  |  |
