# Supplementary material for: Evaluating the role of salt intake in achieving WHO NCD targets in the Eurasian Economic Union: A PRIME modeling study
Source: PLoS One. 2023 Jul 21;18(7):e0289112. doi: 10.1371/journal.pone.0289112 (PMC10361522; doi:10.1371/journal.pone.0289112)
Supplement: S6 Table — (DOCX) [file pone.0289112.s006.docx]

|  | **Males** | | | | | | | | | | | | | | | |
| --- | --- | --- | --- | --- | --- | --- | --- | --- | --- | --- | --- | --- | --- | --- | --- | --- |
| **Age** | **15-19** | **20-24** | **25-29** | **30-34** | **35-39** | **40-44** | **45-49** | **50-54** | **55-59** | **60-64** | **65-69** | **70-75** | **75-79** | **80-84** | **85+** | **Total** |
| I60-I69: Cerebrovascular diseases | 19 | 66 | 134 | 494 | 1010 | 1791 | 2723 | 4037 | 7795 | 12788 | 15761 | 14117 | 13805 | 17169 | 13664 | 105373 |
| I20-I25: Ischaemic heart diseases | 27 | 73 | 235 | 821 | 1725 | 3620 | 6336 | 10711 | 21275 | 31854 | 34142 | 27201 | 24024 | 28658 | 22477 | 213179 |
| C00-C14: Lip, oral cavity and pharynx | 1 | 2 | 8 | 33 | 126 | 282 | 529 | 791 | 1403 | 1634 | 1468 | 763 | 341 | 223 | 95 | 7699 |
| C15: Oesophagus | 0 | 0 | 2 | 10 | 40 | 108 | 220 | 448 | 877 | 1170 | 1197 | 689 | 390 | 273 | 115 | 5539 |
| C16: Stomach | 3 | 5 | 18 | 73 | 149 | 305 | 485 | 829 | 1777 | 2804 | 3215 | 2333 | 1633 | 1555 | 719 | 15903 |
| C34: Bronchus and lung | 2 | 9 | 12 | 45 | 152 | 435 | 962 | 2,178 | 5,324 | 8,658 | 9,485 | 6,162 | 3,439 | 2,491 | 891 | 40245 |
| C25: Pancreas | 0 | 1 | 5 | 33 | 86 | 198 | 394 | 684 | 1322 | 1738 | 1951 | 1333 | 792 | 691 | 323 | 9551 |
| C18-20: Colorectum | 2 | 4 | 24 | 69 | 149 | 264 | 412 | 739 | 1,839 | 2,783 | 3,711 | 2,913 | 2,351 | 2,329 | 1,156 | 18745 |
| C50: Breast | 0 | 0 | 0 | 3 | 2 | 2 | 4 | 5 | 20 | 29 | 23 | 27 | 16 | 14 | 6 | 151 |
| C54.1: Endometrium | N/A | N/A | N/A | N/A | N/A | N/A | N/A | N/A | N/A | N/A | N/A | N/A | N/A | N/A | N/A | 0 |
| C23: Gallbladder | N/A | N/A | N/A | N/A | N/A | N/A | N/A | N/A | N/A | N/A | N/A | N/A | N/A | N/A | N/A | 0 |
| C64: Kidney | N/A | N/A | N/A | N/A | N/A | N/A | N/A | N/A | N/A | N/A | N/A | N/A | N/A | N/A | N/A | 0 |
| I10-I15: Hypertensive disease | 1 | 4 | 9 | 22 | 53 | 96 | 200 | 327 | 614 | 904 | 1,041 | 843 | 828 | 897 | 760 | 6599 |
| E11,E14: Diabetes | 8 | 31 | 54 | 138 | 151 | 247 | 325 | 522 | 989 | 1,818 | 2,427 | 2,143 | 1,420 | 1,553 | 785 | 12611 |
| C67: Bladder cancer | 0 | 0 | 1 | 0 | 17 | 20 | 67 | 150 | 391 | 654 | 921 | 819 | 690 | 720 | 353 | 4803 |
| C22: Liver cancer | 1 | 2 | 9 | 26 | 42 | 138 | 267 | 422 | 840 | 1133 | 1149 | 786 | 538 | 458 | 210 | 6021 |
| C53: Cervix cancer | 0 | 0 | 0 | 0 | 0 | 0 | 0 | 0 | 0 | 0 | 0 | 0 | 0 | 0 | 0 | 0 |
| J40-J44: COPD | 11 | 11 | 20 | 51 | 131 | 214 | 316 | 587 | 1,382 | 2,633 | 3,711 | 3,177 | 2,976 | 3,382 | 2,229 | 20831 |
| K70, K74: Liver disease | 5 | 21 | 235 | 1,076 | 2,280 | 3,189 | 3,313 | 3,101 | 3,831 | 4,057 | 2,946 | 1,381 | 768 | 614 | 276 | 27093 |
| I50: Heart failure | N/A | N/A | N/A | N/A | N/A | N/A | N/A | N/A | N/A | N/A | N/A | N/A | N/A | N/A | N/A | 0 |
| I71: Aortic aneurysm | 0 | 0 | 0 | 2 | 6 | 21 | 35 | 134 | 382 | 788 | 1,267 | 1,101 | 1,063 | 1,261 | 860 | 6920 |
| I26: Pulmonary embolism | N/A | N/A | N/A | N/A | N/A | N/A | N/A | N/A | N/A | N/A | N/A | N/A | N/A | N/A | N/A | 0 |
| I05-09: Rheumatic heart disease | 1 | 1 | 6 | 4 | 16 | 13 | 30 | 50 | 86 | 111 | 135 | 90 | 54 | 43 | 24 | 664 |
| N18: Chronic renal failure | 2 | 7 | 18 | 48 | 84 | 144 | 193 | 256 | 408 | 670 | 745 | 751 | 776 | 919 | 683 | 5704 |
| **Total** | 83 | 237 | 790 | 2,948 | 6,219 | 11,087 | 16,811 | 25,971 | 50,555 | 76,226 | 85,295 | 66,629 | 55,904 | 63,250 | 45,626 | 507,631 |
|  |  |  |  |  |  |  |  |  |  |  |  |  |  |  |  |  |
|  | **Females** | | | | | | | | | | | | | | | |
| **Age** | **15-19** | **20-24** | **25-29** | **30-34** | **35-39** | **40-44** | **45-49** | **50-54** | **55-59** | **60-64** | **65-69** | **70-75** | **75-79** | **80-84** | **85+** | **Total** |
| I60-I69: Cerebrovascular diseases | 12 | 23 | 79 | 210 | 445 | 747 | 1223 | 1768 | 3684 | 6517 | 10669 | 13489 | 21996 | 39475 | 54281 | 154618 |
| I20-I25: Ischaemic heart diseases | 8 | 15 | 61 | 178 | 367 | 768 | 1337 | 2504 | 6056 | 11596 | 18581 | 21595 | 33074 | 56192 | 75707 | 228039 |
| C00-C14: Lip, oral cavity and pharynx | 3 | 2 | 2 | 17 | 41 | 81 | 143 | 144 | 265 | 304 | 286 | 229 | 149 | 212 | 194 | 2072 |
| C15: Oesophagus | 0 | 2 | 1 | 6 | 13 | 22 | 46 | 92 | 125 | 203 | 226 | 165 | 195 | 221 | 159 | 1476 |
| C16: Stomach | 3 | 6 | 29 | 73 | 139 | 216 | 310 | 375 | 781 | 1187 | 1595 | 1523 | 1626 | 2100 | 1364 | 11327 |
| C34: Bronchus and lung | 0 | 6 | 6 | 39 | 77 | 145 | 241 | 446 | 887 | 1,368 | 1,690 | 1,459 | 1,152 | 1,251 | 941 | 9708 |
| C25: Pancreas | 1 | 2 | 7 | 19 | 38 | 100 | 162 | 327 | 733 | 1267 | 1721 | 1496 | 1469 | 1571 | 1096 | 10009 |
| C18-20: Colorectum | 2 | 8 | 16 | 72 | 137 | 231 | 366 | 676 | 1,393 | 2,270 | 3,115 | 2,973 | 3,103 | 3,966 | 2,866 | 21194 |
| C50: Breast | 0 | 6 | 38 | 182 | 450 | 752 | 1114 | 1446 | 2522 | 3122 | 3274 | 2625 | 2107 | 2397 | 1659 | 21694 |
| C54.1: Endometrium | N/A | N/A | N/A | N/A | N/A | N/A | N/A | N/A | N/A | N/A | N/A | N/A | N/A | N/A | N/A | 0 |
| C23: Gallbladder | N/A | N/A | N/A | N/A | N/A | N/A | N/A | N/A | N/A | N/A | N/A | N/A | N/A | N/A | N/A | 0 |
| C64: Kidney | N/A | N/A | N/A | N/A | N/A | N/A | N/A | N/A | N/A | N/A | N/A | N/A | N/A | N/A | N/A | 0 |
| I10-I15: Hypertensive disease | 0 | 0 | 2 | 9 | 15 | 43 | 72 | 150 | 328 | 581 | 855 | 951 | 1,487 | 2,189 | 2,811 | 9493 |
| E11,E14: Diabetes | 12 | 17 | 40 | 71 | 81 | 132 | 181 | 365 | 1,060 | 2,319 | 4,099 | 4,495 | 4,745 | 6,548 | 4,301 | 28466 |
| C67: Bladder cancer | 0 | 0 | 1 | 3 | 8 | 6 | 18 | 27 | 50 | 109 | 144 | 173 | 207 | 325 | 251 | 1322 |
| C22: Liver cancer | 6 | 3 | 8 | 19 | 25 | 62 | 90 | 159 | 307 | 501 | 704 | 566 | 599 | 756 | 572 | 4377 |
| C53: Cervix cancer | 0 | 9 | 64 | 268 | 514 | 617 | 677 | 583 | 804 | 770 | 691 | 426 | 336 | 370 | 253 | 6382 |
| J40-J44: COPD | 7 | 10 | 9 | 25 | 30 | 75 | 86 | 175 | 334 | 571 | 838 | 824 | 1,206 | 1,948 | 2,302 | 8440 |
| K70, K74: Liver disease | 3 | 17 | 167 | 661 | 1,306 | 1,737 | 1,773 | 1,979 | 3,014 | 2,835 | 2,237 | 1,312 | 915 | 950 | 632 | 19538 |
| I50: Heart failure | N/A | N/A | N/A | N/A | N/A | N/A | N/A | N/A | N/A | N/A | N/A | N/A | N/A | N/A | N/A | 0 |
| I71: Aortic aneurysm | 0 | 0 | 0 | 0 | 3 | 6 | 18 | 38 | 124 | 268 | 488 | 713 | 1,164 | 2,283 | 3,135 | 8240 |
| I26: Pulmonary embolism | N/A | N/A | N/A | N/A | N/A | N/A | N/A | N/A | N/A | N/A | N/A | N/A | N/A | N/A | N/A | 0 |
| I05-09: Rheumatic heart disease | 0 | 1 | 2 | 3 | 4 | 6 | 19 | 44 | 109 | 202 | 272 | 248 | 262 | 267 | 192 | 1631 |
| N18: Chronic renal failure | 5 | 7 | 25 | 37 | 78 | 90 | 139 | 207 | 412 | 597 | 887 | 861 | 1,034 | 1,575 | 1,653 | 7607 |
| **Total** | 62 | 134 | 557 | 1,892 | 3,771 | 5,836 | 8,015 | 11,505 | 22,988 | 36,587 | 52,372 | 56,123 | 76,826 | 124,596 | 154,369 | 555,633 |
|  |  |  |  |  |  |  |  |  |  |  |  |  |  |  |  |  |
