# Supplementary material for: Evaluating the role of salt intake in achieving WHO NCD targets in the Eurasian Economic Union: A PRIME modeling study
Source: PLoS One. 2023 Jul 21;18(7):e0289112. doi: 10.1371/journal.pone.0289112 (PMC10361522; doi:10.1371/journal.pone.0289112)
Supplement: S7 Table — (DOCX) [file pone.0289112.s007.docx]

|  | **Cerebrovascular Disease**  **(I60-I69)** | | **Coronary Heart Disease**  **(I20-I25)** | | **Hypertensive Heart Disease**  **(I10-I15)** | | **Heart failure**  **(I50)** | | **Aortic aneurysm**  **(I71)** | | **Pulmonary embolism**  **(I26)** | | **Rheumatic heart disease**  **(I05-I09)** | |
| --- | --- | --- | --- | --- | --- | --- | --- | --- | --- | --- | --- | --- | --- | --- |
|  | **Female** | **Male** | **Female** | **Male** | **Female** | **Male** | **Female** | **Male** | **Female** | **Male** | **Female** | **Male** | **Female** | **Male** |
| **Armenia**  (2018) | 68 | 86 | 162 | 266 | 103 | 89 | 22 | 17 | 6 | 14 | 2 | 3 | 1 | 0 |
| **Belarus**  (2018) | 547 | 686 | 1,471 | 2,129 | 11 | 19 | N/A | | 12 | 39 | 0 | 1 | 5 | 3 |
| **Kazakhstan**  (2017) | 786 | 948 | 476 | 835 | 109 | 123 | 69 | 86 | 11 | 26 | 12 | 17 | 9 | 7 |
| **Kyrgyzstan**  (2016) | 262 | 339 | 549 | 592 | 51 | 58 | 5 | 7 | 1 | 1 | 2 | 5 | 5 | 4 |
| **Russia**  (2019) | 17,127 | 14,611 | 21,436 | 23,385 | 2,631 | 1,829 | N/A | | 994 | 835 | N/A | | 102 | 42 |
| **Total** | **18,790** | **16,670** | **24,094** | **27,207** | **2,905** | **2,118** | **96** | **110** | **1,024** | **915** | **16** | **26** | **122** | **56** |
|  | **35,460** | | **51,301** | | **5,023** | | **206** | | **1,939** | | **42** | | **178** | |
