# Supplementary material for: Evaluating the role of salt intake in achieving WHO NCD targets in the Eurasian Economic Union: A PRIME modeling study
Source: PLoS One. 2023 Jul 21;18(7):e0289112. doi: 10.1371/journal.pone.0289112 (PMC10361522; doi:10.1371/journal.pone.0289112)
Supplement: S8 Table — (DOCX) [file pone.0289112.s008.docx]

|  | **Cerebrovascular Disease**  **(I60-I69)** | | **Coronary Heart Disease**  **(I20-I25)** | | **Hypertensive Heart Disease**  **(I10-I15)** | | **Heart failure**  **(I50)** | | **Aortic aneurysm**  **(I71)** | | **Pulmonary embolism**  **(I26)** | | **Rheumatic heart disease**  **(I05-I09)** | |
| --- | --- | --- | --- | --- | --- | --- | --- | --- | --- | --- | --- | --- | --- | --- |
|  | **Female** | **Male** | **Female** | **Male** | **Female** | **Male** | **Female** | **Male** | **Female** | **Male** | **Female** | **Male** | **Female** | **Male** |
| **Armenia**  (2018) | 90 | 149 | 218 | 474 | 134 | 148 | 29 | 30 | 9 | 24 | 3 | 5 | 1 | 1 |
| **Belarus**  (2018) | 792 | 1,278 | 2,172 | 4,101 | 16 | 33 | N/A | | 18 | 74 | 1 | 1 | 6 | 7 |
| **Kazakhstan**  (2017) | 1,716 | 2,190 | 1,080 | 2,016 | 219 | 264 | 152 | 203 | 26 | 61 | 27 | 42 | 20 | 18 |
| **Kyrgyzstan**  (2016) | 569 | 786 | 1,248 | 1,437 | 103 | 124 | 10 | 17 | 2 | 3 | 6 | 12 | 12 | 9 |
| **Russia**  (2019) | 35,083 | 29,443 | 45,207 | 48,921 | 4,865 | 3,383 | N/A | | 2,043 | 1,715 | N/A | | 218 | 89 |
| **Total** | **38,250** | **33,846** | **49,925** | **56,949** | **5,337** | **3,952** | **191** | **250** | **2,098** | **1,877** | **37** | **60** | **257** | **124** |
|  | **72,096** | | **106,874** | | **9,289** | | **441** | | **3,975** | | **97** | | **381** | |
