# Supplementary material for: Evaluating the role of salt intake in achieving WHO NCD targets in the Eurasian Economic Union: A PRIME modeling study
Source: PLoS One. 2023 Jul 21;18(7):e0289112. doi: 10.1371/journal.pone.0289112 (PMC10361522; doi:10.1371/journal.pone.0289112)
Supplement: S9 Table — (DOCX) [file pone.0289112.s009.docx]

| **Female** | | | | | | | | | | | | | | | | |
| --- | --- | --- | --- | --- | --- | --- | --- | --- | --- | --- | --- | --- | --- | --- | --- | --- |
|  | **15-19** | **20-24** | **25-29** | **30-34** | **35-39** | **40-44** | **45-49** | **50-54** | **55-59** | **60-64** | **65-69** | **70-74** | **75-79** | **80-84** | **85+** | **Total** |
| **Armenia** | 0 | 0 | 1 | 0 | 1 | 1 | 3 | 6 | 15 | 22 | 31 | 29 | 66 | 81 | 107 | 364 |
| **Belarus** | 0 | 0 | 1 | 2 | 5 | 11 | 18 | 33 | 79 | 120 | 190 | 192 | 405 | 398 | 594 | 2047 |
| **Kazakhstan** | 1 | 1 | 2 | 8 | 16 | 27 | 47 | 74 | 123 | 144 | 206 | 132 | 320 | 177 | 193 | 1472 |
| **Kyrgyzstan** | 1 | 1 | 1 | 2 | 4 | 11 | 17 | 30 | 57 | 76 | 95 | 70 | 171 | 134 | 204 | 875 |
| **Russia** | 3 | 7 | 24 | 68 | 142 | 264 | 444 | 714 | 1611 | 2693 | 4335 | 4444 | 7001 | 8730 | 11811 | 42290 |
| **EEU** | 6 | 9 | 29 | 81 | 167 | 314 | 528 | 857 | 1884 | 3055 | 4858 | 4867 | 7963 | 9520 | 12909 | 47047 |

| **Male** | | | | | | | | | | | | | | | | |
| --- | --- | --- | --- | --- | --- | --- | --- | --- | --- | --- | --- | --- | --- | --- | --- | --- |
|  | **15-19** | **20-24** | **25-29** | **30-34** | **35-39** | **40-44** | **45-49** | **50-54** | **55-59** | **60-64** | **65-69** | **70-74** | **75-79** | **80-84** | **85+** | **Total** |
| **Armenia** | 0 | 1 | 1 | 3 | 4 | 6 | 13 | 29 | 54 | 57 | 67 | 43 | 69 | 62 | 68 | 475 |
| **Belarus** | 0 | 1 | 3 | 12 | 24 | 49 | 98 | 162 | 318 | 434 | 495 | 338 | 396 | 286 | 260 | 2877 |
| **Kazakhstan** | 2 | 4 | 10 | 19 | 41 | 73 | 129 | 207 | 316 | 307 | 332 | 175 | 254 | 103 | 71 | 2043 |
| **Kyrgyzstan** | 0 | 0 | 3 | 6 | 12 | 24 | 44 | 72 | 119 | 137 | 142 | 73 | 164 | 103 | 109 | 1006 |
| **Russia** | 8 | 24 | 61 | 215 | 448 | 872 | 1454 | 2297 | 4523 | 6334 | 7202 | 5110 | 4732 | 4151 | 3272 | 40701 |
| **EEU** | 10 | 30 | 78 | 255 | 529 | 1023 | 1738 | 2766 | 5329 | 7269 | 8238 | 5739 | 5614 | 4706 | 3779 | 47102 |
