# Supplementary material for: Evaluating the role of salt intake in achieving WHO NCD targets in the Eurasian Economic Union: A PRIME modeling study
Source: PLoS One. 2023 Jul 21;18(7):e0289112. doi: 10.1371/journal.pone.0289112 (PMC10361522; doi:10.1371/journal.pone.0289112)
Supplement: S10 Table — (DOCX) [file pone.0289112.s010.docx]

| **Female** | | | | | | | | | | | | | | | | |
| --- | --- | --- | --- | --- | --- | --- | --- | --- | --- | --- | --- | --- | --- | --- | --- | --- |
|  | **15-19** | **20-24** | **25-29** | **30-34** | **35-39** | **40-44** | **45-49** | **50-54** | **55-59** | **60-64** | **65-69** | **70-74** | **75-79** | **80-84** | **85+** | **Total** |
| **Armenia** | 0 | 0 | 1 | 1 | 1 | 2 | 4 | 8 | 20 | 30 | 41 | 39 | 88 | 108 | 143 | 485 |
| **Belarus** | 0 | 0 | 1 | 3 | 7 | 16 | 26 | 48 | 115 | 175 | 278 | 282 | 594 | 587 | 875 | 3007 |
| **Kazakhstan** | 2 | 3 | 5 | 18 | 35 | 58 | 100 | 160 | 265 | 315 | 451 | 291 | 704 | 399 | 434 | 3240 |
| **Kyrgyzstan** | 3 | 2 | 2 | 4 | 8 | 24 | 37 | 66 | 125 | 167 | 210 | 155 | 381 | 303 | 464 | 1951 |
| **Russia** | 7 | 13 | 49 | 136 | 284 | 528 | 892 | 1438 | 3250 | 5470 | 8809 | 9126 | 14370 | 18290 | 24755 | 87417 |
| **EEU** | **11** | **18** | **58** | **162** | **335** | **628** | **1059** | **1720** | **3775** | **6157** | **9788** | **9892** | **16137** | **19687** | **26671** | **96100** |

| **Male** | | | | | | | | | | | | | | | | |
| --- | --- | --- | --- | --- | --- | --- | --- | --- | --- | --- | --- | --- | --- | --- | --- | --- |
|  | **15-19** | **20-24** | **25-29** | **30-34** | **35-39** | **40-44** | **45-49** | **50-54** | **55-59** | **60-64** | **65-69** | **70-74** | **75-79** | **80-84** | **85+** | **Total** |
| **Armenia** | 0 | 1 | 2 | 6 | 6 | 11 | 22 | 50 | 94 | 99 | 118 | 74 | 120 | 109 | 119 | 831 |
| **Belarus** | 0 | 2 | 5 | 23 | 46 | 93 | 186 | 308 | 603 | 824 | 940 | 646 | 757 | 555 | 505 | 5493 |
| **Kazakhstan** | 5 | 10 | 24 | 44 | 94 | 169 | 301 | 482 | 736 | 719 | 776 | 415 | 600 | 249 | 170 | 4795 |
| **Kyrgyzstan** | 0 | 1 | 7 | 13 | 28 | 55 | 104 | 167 | 277 | 322 | 333 | 173 | 390 | 251 | 265 | 2387 |
| **Russia** | 16 | 48 | 123 | 434 | 906 | 1767 | 2952 | 4665 | 9192 | 12932 | 14685 | 10524 | 9733 | 8711 | 6862 | 83550 |
| **EEU** | **21** | **62** | **160** | **520** | **1081** | **2096** | **3566** | **5672** | **10902** | **14896** | **16852** | **11833** | **11600** | **9875** | **7920** | **97056** |
