# Supplementary material for: Evaluating the role of salt intake in achieving WHO NCD targets in the Eurasian Economic Union: A PRIME modeling study
Source: PLoS One. 2023 Jul 21;18(7):e0289112. doi: 10.1371/journal.pone.0289112 (PMC10361522; doi:10.1371/journal.pone.0289112)
Supplement: S11 Table — (DOCX) [file pone.0289112.s011.docx]

| **ICD-10 Code** | **Cardiovascular Disease Name** |
| --- | --- |
| I60-I69 | Cerebrovascular diseases (Stroke) |
| I20-I25 | Ischaemic heart diseases (Coronary Heart Disease) |
| I10-I15 | Hypertensive heart disease |
| I50 | Heart failure |
| I71 | Aortic aneurysm |
| I26 | Pulmonary embolism |
| I05-I09 | Rheumatic heart disease |
